# Supplementary material for: A live biohybrid bacterial therapy based on engineered Serratia marcescens
Source: Nat Commun. 2026 Apr 7;17:4956. doi: 10.1038/s41467-026-70949-4 (PMC13234118; doi:10.1038/s41467-026-70949-4)
Supplement: Supplementary file 1 — Supplementary Information [file 41467_2026_70949_MOESM1_ESM.pdf]

Supplementary information of

A live biohybrid bacterial therapy based on engineered *Serratia marcescens*

LihaoJi[1], Tianze Zhu[1], Tianqi Jiang[1], Li Wang[1], Zhonghui Qiu[1], Shiqi Gao[1], Yuqi Wang[1], Jing Wang[2], Jingyi Zhang[2], Haomiao Huang[6], Yunlong Mao[5], Chen Lin[2], Jing Zhao[2,3,4,*], Xiuxiu Wang[1,2,4,**], Wei Wei[1,3,4,***]

[1] State Key Laboratory of Coordination Chemistry, Chemistry and Biomedicine Innovation Center (ChemBIC), School of Life Sciences, Nanjing University, Nanjing 210093, P. R. China.

[2] School of Chemistry, Nanjing University, Nanjing 210093, P. R. China.

[3] Nanchuang (Jiangsu) Institute of Chemistry and Health, Sino-Danish Ecolife Science Industrial Incubator, Jiangbei New Area, Nanjing 210000, P. R. China.

[4] Wuxi Xishan NJU Institute of Applied Biotechnology, Wuxi 214000, P. R. China.

[5] State Key Laboratory of Novel Software Technology, Nanjing University, Nanjing 210093, P. R. China.

[6] Nanjing Foreign Language School, Nanjing 210008, P. R. China.

Present address: School of Life Sciences, Nanjing University, Nanjing 210093, P. R. China.

Lead Contact

*Correspondence: jingzhao@nju.edu.cn

**Correspondence: wangxiuxiu@nju.edu.cn

***Correspondence: weiwei@nju.edu.cn

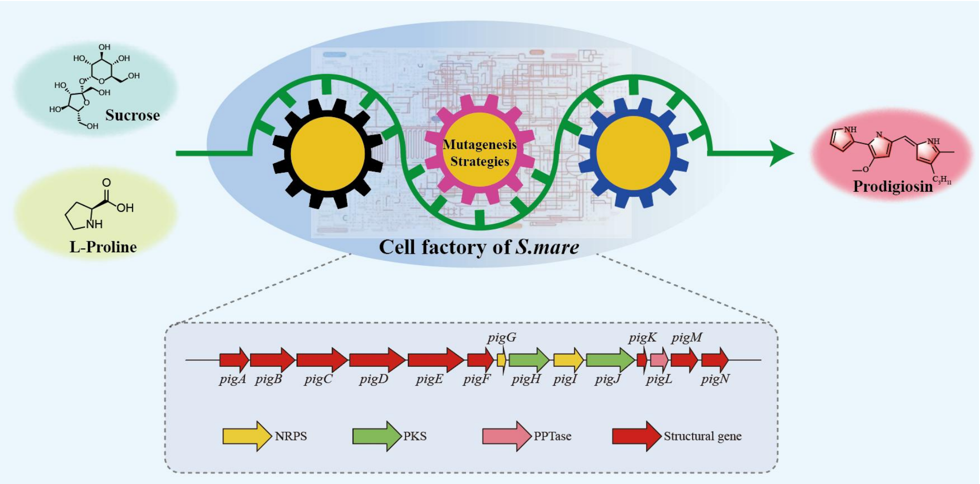

Supplementary Fig 1. Schematic representation of an integrated “cell factory” approach to enhance prodigiosin production in *Serratia marcescens*. Sucrose and *L*-proline serve as the principal carbon and nitrogen sources, respectively. The lower inset depicts the pig gene cluster, highlighting nonribosomal peptide synthetase (NRPS), polyketide synthase (PKS), phosphopantetheinyl transferase (PPTase), and structural genes essential for prodigiosin biosynthesis. All elements in this figure, including chemical structures and schematics, were independently created by the authors, who hold the full copyright. No external images or databases were used.

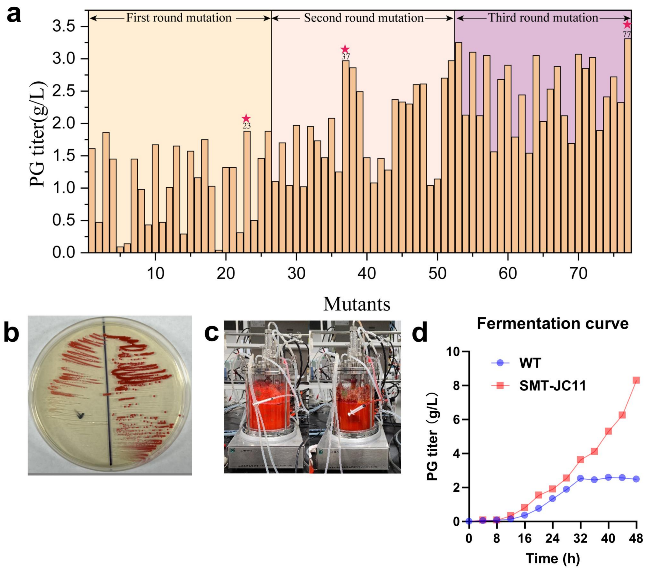

Supplementary Fig 2. Iterative Mutagenesis Yields the Prodigiosin Hyper-producer SM-JC11.

(a) Three rounds of UV mutagenesis and screening, starting from the wild-type (WT), led to the isolation of the high-yield strain SM-JC11.

(b, c) The mutant SM-JC11 (right) displayed a dramatically enhanced red phenotype compared to the WT (left) both on (b) agar plates and in (c) 5-L fermenters.

(d) Fermentation analysis shows that SM-JC11 achieved a significantly higher final Prodigiosin titer compared to the WT. Data represent the mean of three independent experiments (n=3).

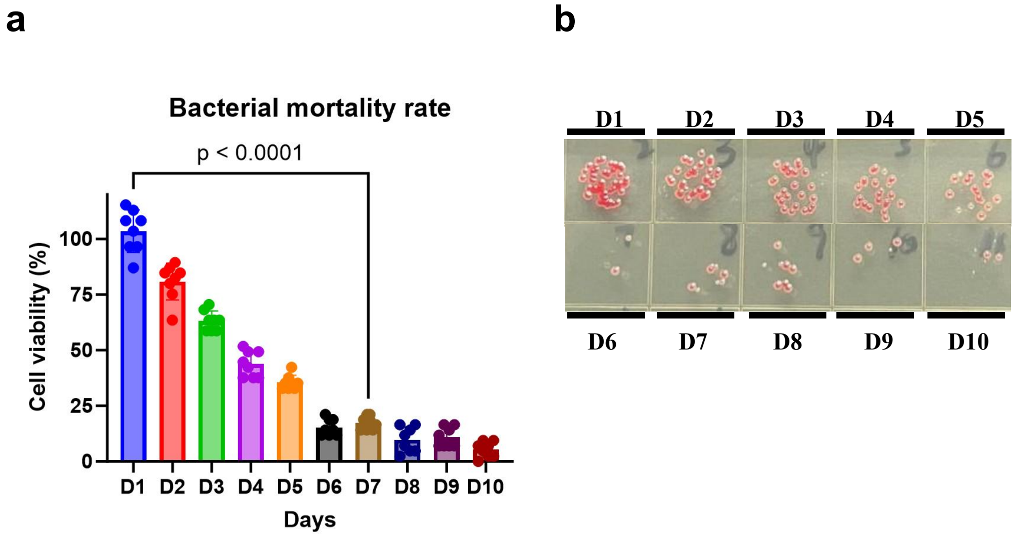

Supplementary Fig 3. (a) Time-dependent changes in bacterial viability (initial concentration 1×1081×108 CFU/mL) under physiological temperature (37 ℃). A gradual decline in cell survival is observed from Day 1 to Day 7, with minimal viability beyond Day 7. Data are presented as mean ± SD of n=8 biologically independent samples. Statistical significance between Day 1 and Day 7 was determined by a two-sided unpaired Student's t-test. Exact *P* values are indicated within the figure. (b) Representative colony morphology over the same ten-day period, demonstrating progressive cellular breakdown and structural deterioration under these temperature conditions.

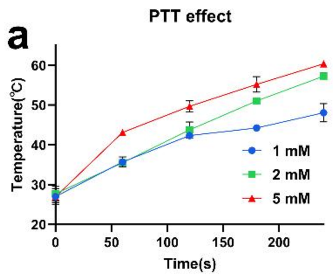

Supplementary Fig 4. (a) Photothermal heating profiles of samples at three concentrations (1 mM, 2 mM, 5 mM) during continuous 808 nm irradiation, showing distinct temperature elevations over time.

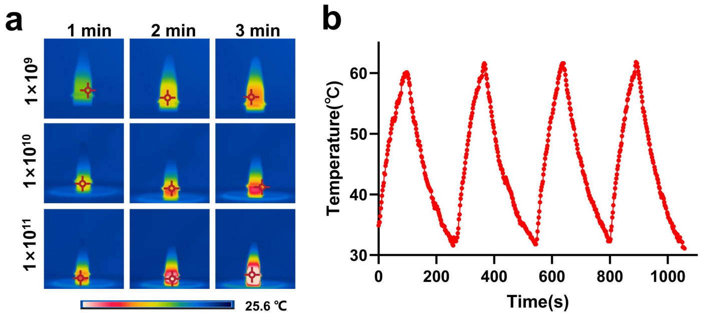

Supplementary Fig 5. Photothermal performance of SM.

(a) Representative infrared thermal images of aqueous suspensions of *Serratia marcescens* JC11 at different concentrations (1×10⁹, 1×10¹⁰, and 1×10¹¹ CFU/mL) under continuous 808 nm laser irradiation (4.5 W/cm²). The results demonstrate a rapid temperature increase that is dependent on both bacterial concentration and irradiation time.

(b) Photothermal stability evaluation of SM suspension (1×10¹¹ CFU/mL) subjected to four successive on/off cycles of 808 nm laser irradiation. The consistent peak temperatures reached in each cycle confirm the excellent photostability and reliable heating performance of the bacterial agent.

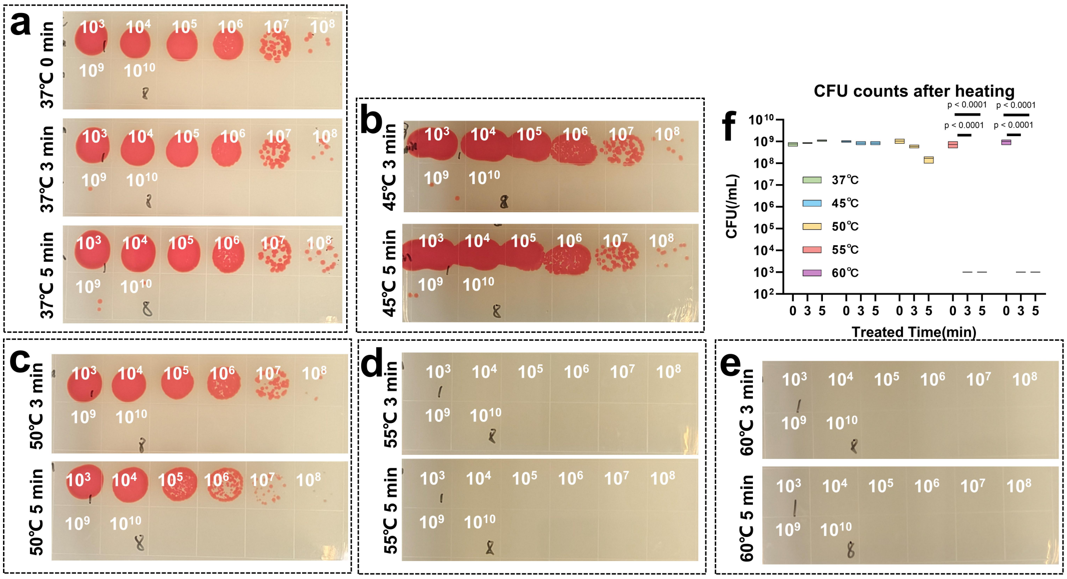

Supplementary Fig 6 Effect of temperature and exposure time on bacterial survival (spot-plate assay).

(a–e) Representative spot plates of log-phase *Serratia marcescens* JC11 exposed to 37℃, 45℃, 50℃, 55℃, or 60℃ for 0, 3, or 5 min, as indicated. Immediately after heating, samples were chilled on ice, subjected to 10-fold serial dilutions, and 10 μL of each dilution (10^3–10^{10}) was spotted onto agar and incubated at 37℃ overnight. The starting culture was approximately 1×10^9 CFU/mL. Survival was unchanged at 37℃; 45℃

produced a modest reduction (5 min); 50℃ markedly decreased viability; 55℃ yielded near-complete killing by 5 min; and at 60℃ no colonies were detectable within 3–5 min. (f) Viable counts (CFU/mL) calculated from countable spots under the indicated conditions. Colors denote temperatures and the x-axis shows treatment time (0, 3, 5 min).

33

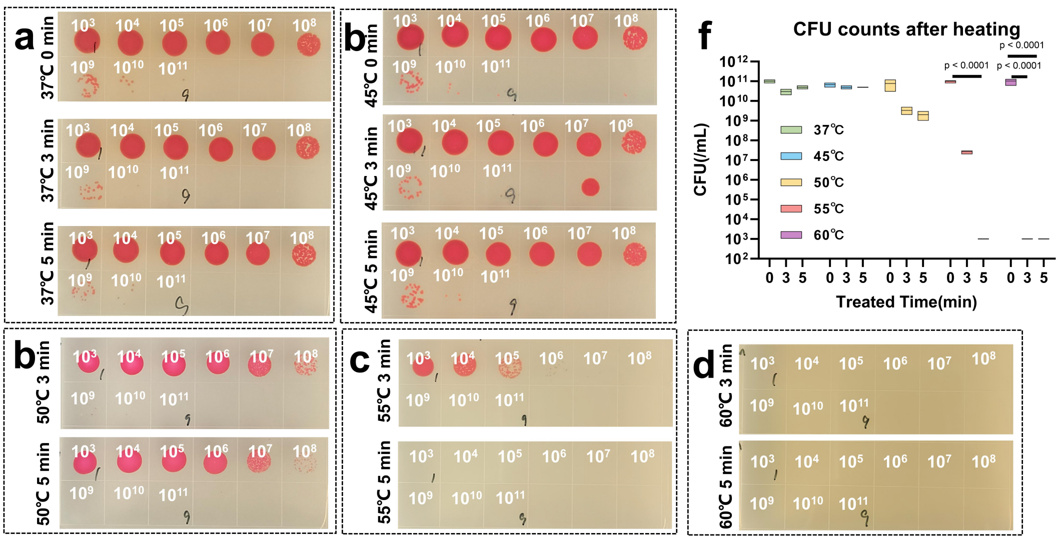

Supplementary Fig 7 Effect of temperature and exposure time on bacterial survival (spot-plate assay).

(a–e) Representative spot plates of log-phase SM exposed to 37℃, 45℃, 50℃, 55℃, or 60℃ for 0, 3, or 5 min, as indicated. Immediately after heating, samples were chilled on ice, subjected to 10-fold serial dilutions, and 6 μL of each dilution (10^3–10^{10}) was spotted onto agar and incubated at 37℃ overnight. The starting culture was approximately $1×10^{11}$ CFU/mL. Survival was unchanged at 37℃; 45℃ produced a modest reduction (5 min); 50℃ markedly decreased viability; 55℃ yielded near-complete killing by 5 min; and at 60℃ no colonies were detectable within 3–5 min.

(f) Viable counts (CFU/mL) calculated from countable spots under the indicated conditions. Colors denote temperatures and the X-axis shows treatment time (0, 3, 5 min).

34

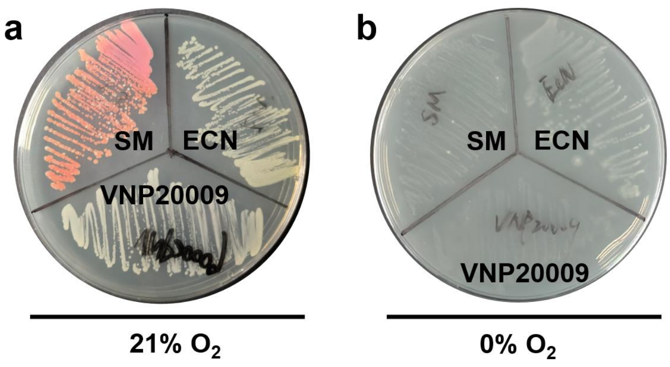

Supplementary Fig 8 Growth of SM, ECN, and VNP2000 on LB agar plates following incubation at 37℃ for 48 hours under aerobic (21% O_2, panel a) and anaerobic (0% O_2, panel b) conditions. SM exhibits its characteristic red pigmentation under aerobic conditions but exhibits significantly reduced growth under anaerobic conditions.

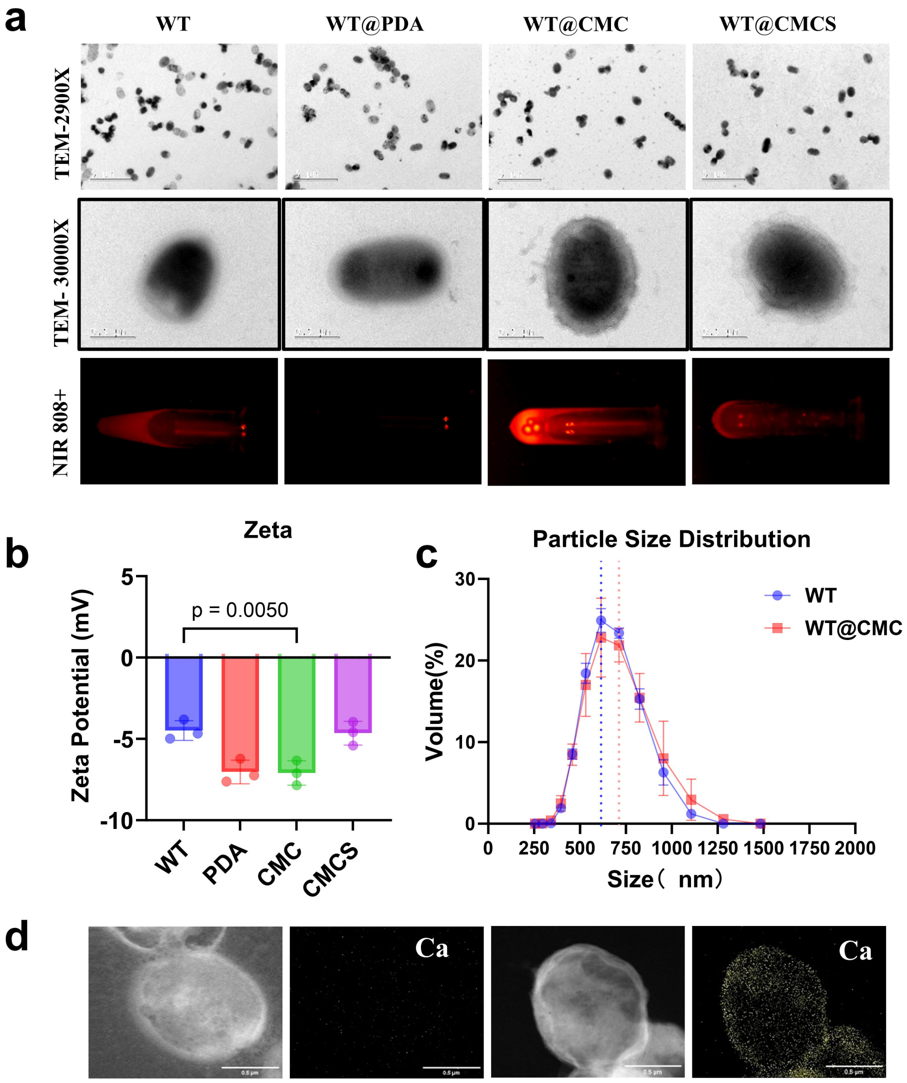

Supplementary Fig 9. (a) Representative TEM and near-infrared fluorescence images acquired for wild-type *S. marcescens*(WT), WT@PDA, WT@CMC, and WT@CMCS, demonstrating distinct morphological and optical features after each modification. (b) Zeta potential measurements of WT, PDA, CMC, and CMCS strains, reflecting charge variations upon surface coating. (c) Particle size distribution profiles (by volume) for WT and WT@CMC, obtained via dynamic light scattering. Data are presented as mean ± SD of n=3 biologically independent samples. Statistical significance between WT and CMC was determined by by a two-sided unpaired Student's t-test. Exact *P* values are indicated within the figure. (d) EDS scanning images confirming the elemental distribution in *S. marcescens* after Ca^{2+} doping and CMC functionalization.

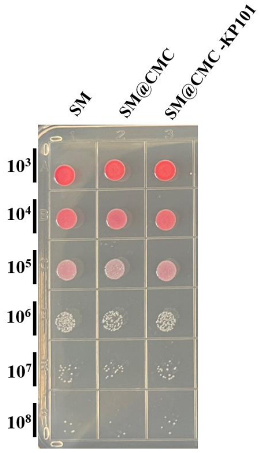

Supplementary Fig 10. Spot plate assays evaluating the stability of SM, SM@ CMC, and SM CMC - KP101. Overnight cultures were serially diluted (10^1 to 10^8) and spotted onto LB agar.

37

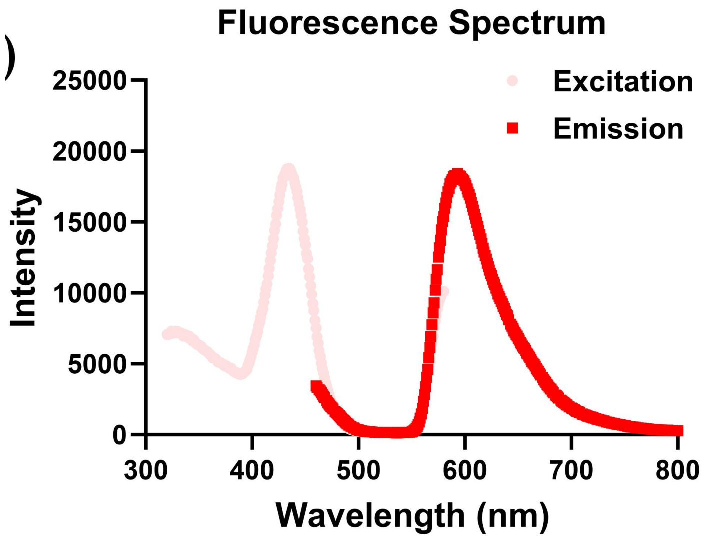

Supplementary Fig 11. The excitation (light pink circles) and emission (red squares) intensity profiles across a wavelength range of 300 to 800 nm.

38

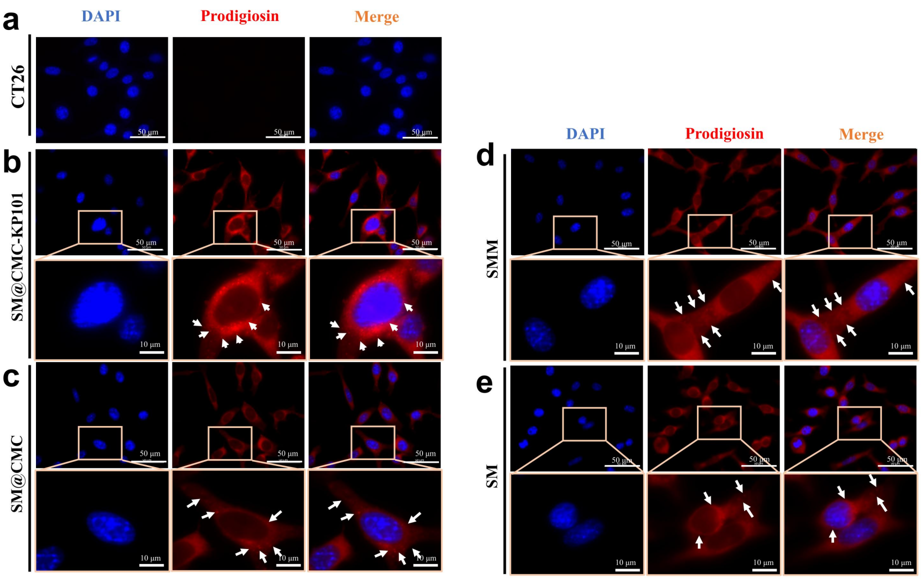

Supplementary Fig 12. Internalization of *S. marcescens* formulations in CT26 cells. Fluorescence microscopy images taken after a 1-hour incubation and subsequent amikacin treatment to remove extracellular bacteria. Panels show control cells (a) and cells treated with SM@CMC-KP101 (b), SM@CMC (c), SMM (d), and SM (e). Red: Prodigiosin (bacteria). Blue: DAPI (nuclei). White arrows indicate internalized bacteria. Scale bars are 50 μm and 10 μm (insets).

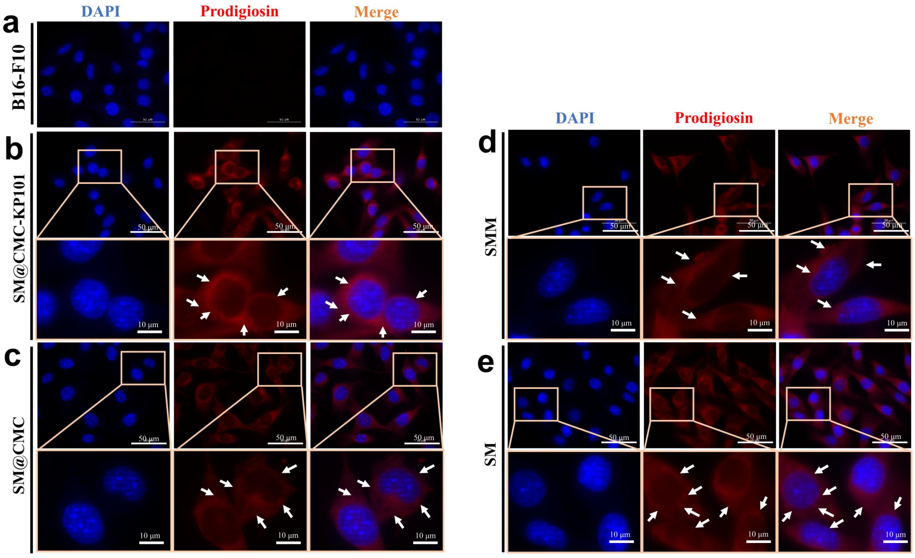

Supplementary Fig 13. Internalization of *S. marcescens* formulations in B16F10 cells. Fluorescence microscopy images taken after a 1-hour incubation and subsequent amikacin treatment to remove extracellular bacteria. Panels show control cells (a) and cells treated with SM@CMC-KP101 (b), SM@CMC (c), SMM (d), and SM (e). Red: Prodigiosin (bacteria). Blue: DAPI (nuclei). White arrows indicate internalized bacteria. Scale bars are 50 μm and 10 μm (insets).

40

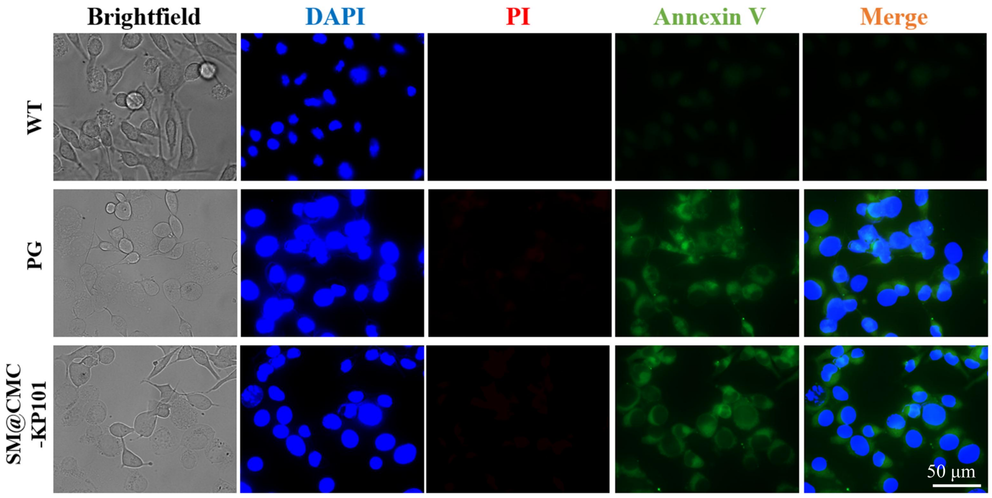

Supplementary Fig 14. B16F10 cells imaged in brightfield and stained with DAPI (blue), PI (red), and Annexin V (green) to evaluate apoptosis. PBS-treated cells display minimal PI and Annexin V signals. Upon PG treatment, significant PI and Annexin V positivity is observed, indicative of enhanced apoptotic and/or necrotic cell death. Co-treatment with SM@CMC-KP101 and PG further increases these apoptotic markers, as visualized by the merged fluorescence images.Scale bars are 50 μm.

41

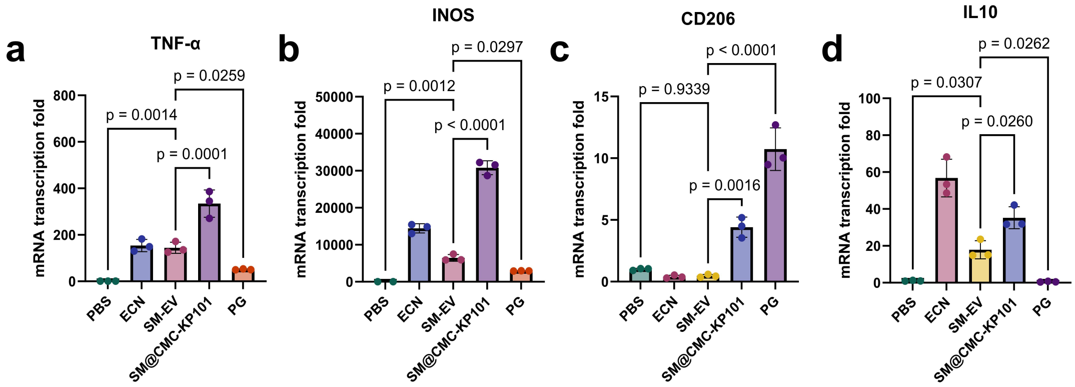

Supplementary Fig 15. The qPCR analysis of TNF-α (a), iNOS (b), CD206 (c), and IL-10 (d) mRNA expression in bone marrow–derived dendritic cells (BMDCs) treated with PBS, ECN, SM-EV, SM@CMC-KP101, or PG (n=3). Data are presented as mean ± SD of n=3 biologically independent samples per group. Statistical significance was determined by a two-sided ordinary one-way ANOVA followed by Tukey's multiple comparisons test. Exact *P* values are indicated within the figure.

42

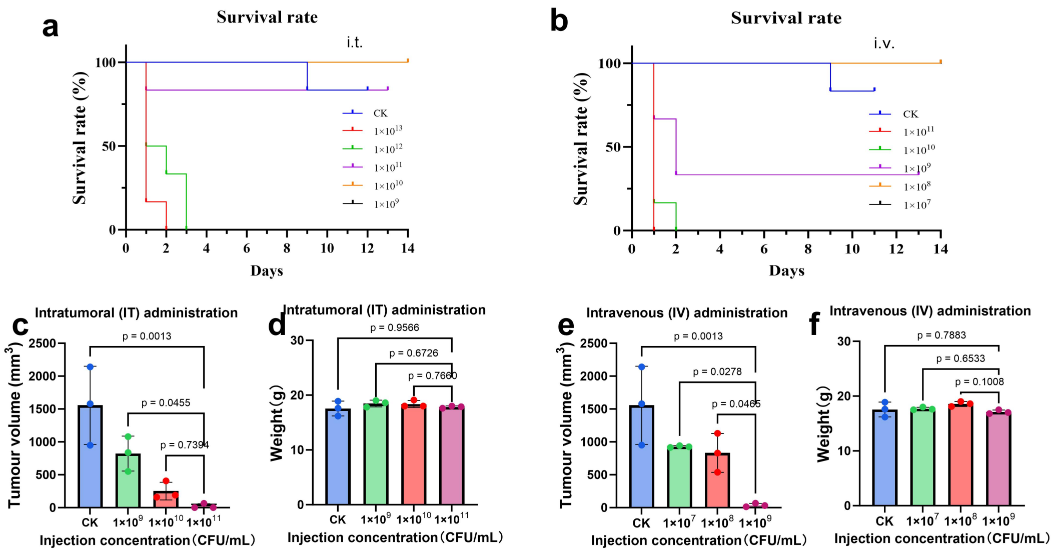

Supplementary Fig 16. Determination of optimal therapeutic dosage by evaluating dose-dependent safety and efficacy of engineered bacteria in CT26 tumor-bearing mice.

(a, b) Dose-escalation studies to determine the Maximum Tolerated Dose (MTD). Kaplan-Meier survival curves of Balb/c mice bearing CT26 tumors (n = 5 per group) following a single injection of bacteria at the indicated concentrations via (a) intratumoral (*i.t.*) or (b) intravenous (*i.v.*) routes. The highest i.t. dose ($1×10^{13}$ and $1×10^{12}$ CFU/mL) and i.v. doses ($1×10^{11}$, $1×10^{10}$, and $1×10^{9}$ CFU/mL) exhibited acute toxicity.

(c) Tumor volume and (d) body weight of mice on day 14 after a single IT injection with bacteria at concentrations of $1×10^{11}$, $1×10^{10}$, and $1×10^{9}$ CFU/mL (in a 100 μL volume) (n=3).

(e) Tumor volume and (f) body weight of mice on day 14 after three IV injection at Day 3,6,10 with bacteria at concentrations of $1×10^{7}$, $1×10^{8}$, or $1×10^{9}$ CFU/mL (in a 100 μL volume) (n=3). Data in c-f are presented as mean ± SD of n=3 biologically independent animals per group. Statistical significance was determined by a two-sided ordinary one-way ANOVA followed by Tukey's multiple comparisons test. Exact P values are indicated within the figure.

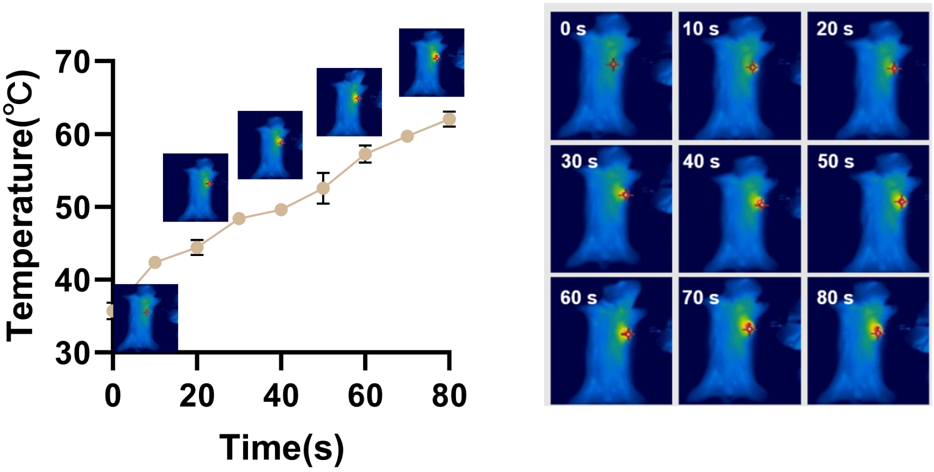

Supplementary Fig 17.Temperature changes over time in CT26 tumors under 808 nm laser irradiation.

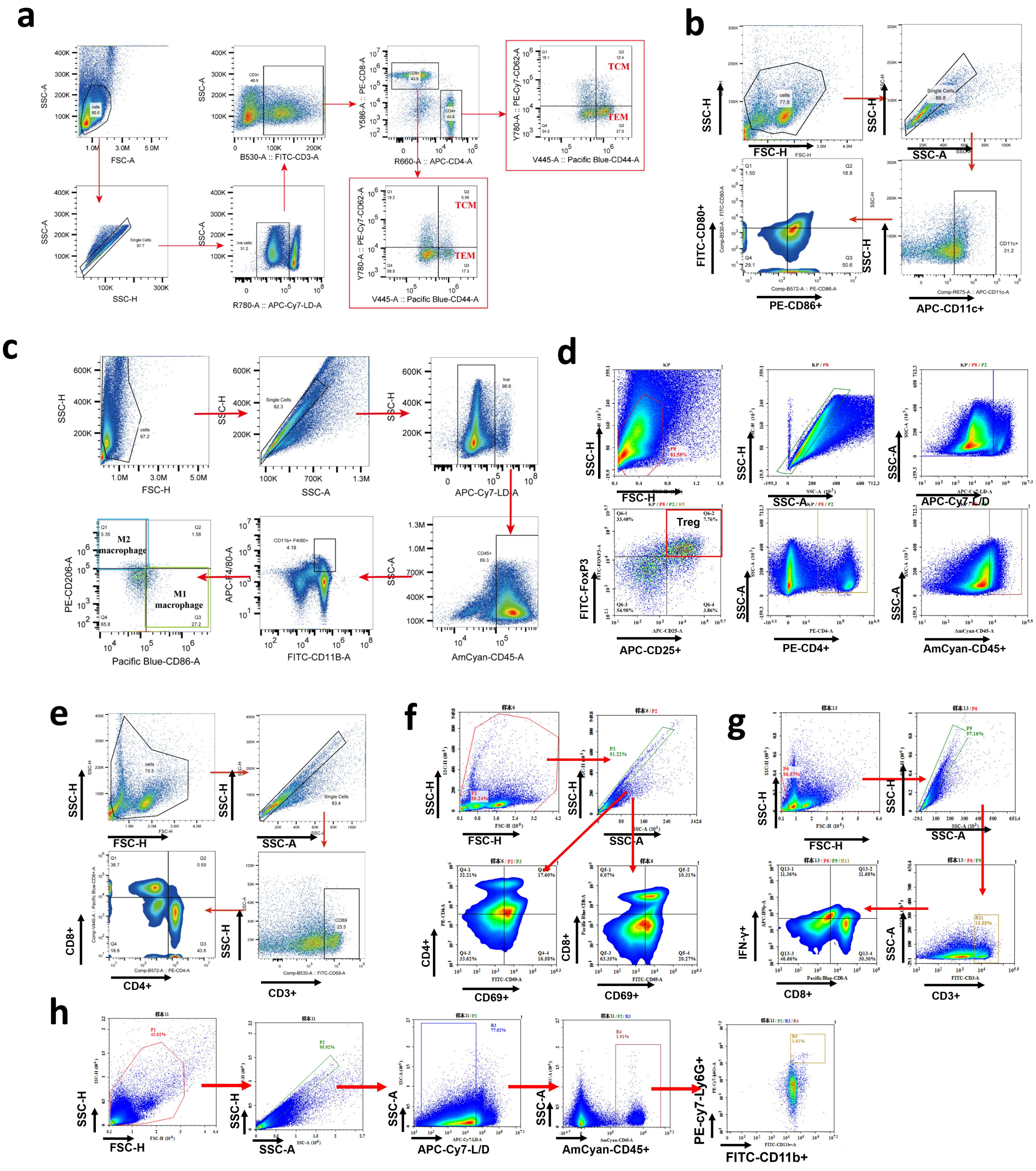

Supplementary Fig 18. Representative flow cytometry gating strategy for immune cell phenotyping. General gating workflow: Cells were initially gated on lymphocytes based on forward scatter (FSC) and side scatter (SSC) characteristics, followed by doublet exclusion using FSC-H/FSC-A and SSC-H/SSC-A plots. Red arrows indicate the sequential gating path. Sample sources and specific staining details are described in the Methods section.

(a) Splenocyte-derived T cell subsets: Live $CD45^+$ cells were gated on $CD3^+$ T cells, then subdivided into $CD4^+$ and $CD8^+$ populations. Memory phenotypes were defined as central memory (TCM:$CD44^+CD62L^+$) and effector memory (TEM: $CD44^+$ $CD62L^-$).

(b) Lymph node-derived dendritic cell (DC) subsets: CD11c+ cells were gated on single cells, with activation status evaluated by CD80 and CD86 co-expression.

(c) Bone marrow-derived macrophage subsets: Live $CD45^+$ cells were gated on $CD11b^+$ F4/80+ macrophages, with polarization assessed by CD86 (M1-like) and CD206 (M2-like) expression.

(d) Splenocyte-derived Treg cells: Live $CD45^+$ cells were first gated on singlets, then on

CD4$^+$ T cells. Regulatory T cells (Treg) were defined as CD25$^+$ FoxP3$^+$ cells within the CD4$^+$ population.

(e) Lymph node-derived total T cell subsets: CD3+ cells were gated on single cells, then separated into CD4+ and CD8+ populations.

(f) Lymph node-derived early-activated T cell subsets: Early activation was assessed by directly gating CD69+CD4+ and CD69+CD8+ T cells using CD69 versus CD4 or CD69 versus CD8 dual-parameter plots.

(g) Splenocyte-derived cytotoxic T cell function: After gating on singlets and CD3+ T cells, CD8+ T cells were identified and further analyzed for intracellular IFN-γ expression following stimulation.

(h) Gating strategy for Neutrophils: After excluding debris and doublets, live CD45+ leukocytes were identified. Neutrophils were then defined and gated as CD11b+Ly6G+ cells within the leukocyte population.

46

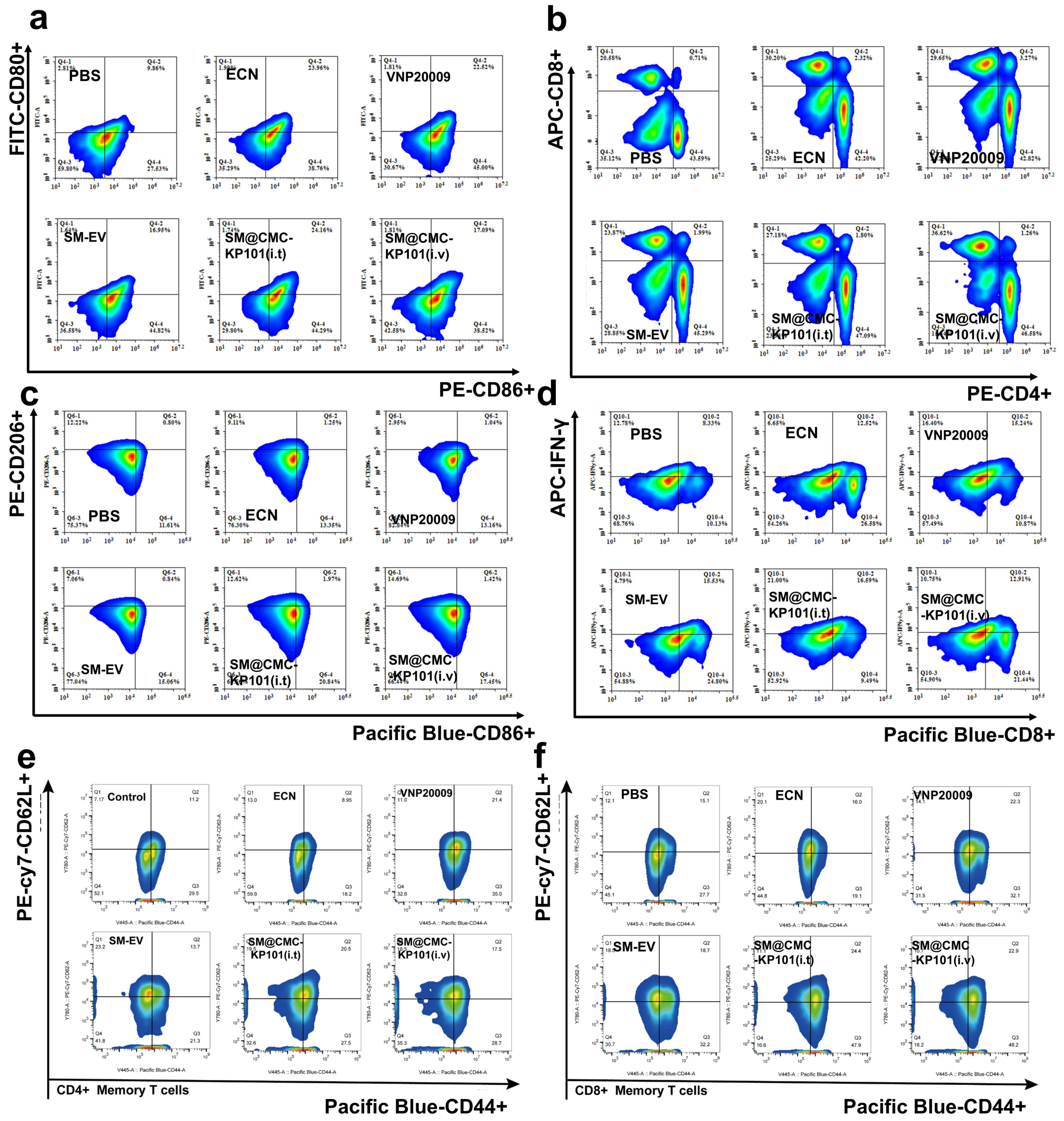

Supplementary Fig 19. Representative Flow Cytometry Analysis of CT26 Tumor-Bearing Mice after Intratumoral Injection. (a) The impact of different treatments (PBS, ECN,

VNP20009, SM-EV, SM@CMC-KP101(i.t)+NIR, SM@CMC-KP101(i.v)) on the expression of CD80^{+} and CD86^{+} on gated CD11c^{+} dendritic cells (DCs) from tumor-draining lymph nodes. (b) Flow cytometry dot plots showing CD8^{+} and CD4^{+} T cell populations on gated CD3^{+} dendritic cells (DCs) from tumor-draining lymph nodes, 24 hours post-treatment. (c) Flow cytometry dot plots showing the expression of CD206^{+} and CD86^{+} on gated bone marrow-derived macrophages, 24 hours post-treatment in vitro. (d) Flow cytometry dot plots showing IFN-γ expression in gated CD8+ T cells from the spleen at the end of the treatment period. (e) Flow cytometry dot plots showing CD44 and CD62L expression on gated CD4+ T cells from the spleen at the end of the treatment period. (f) Flow cytometry dot plots showing CD44 and CD62L expression on gated CD8+ T cells from the spleen at the end of the treatment period. Treatments included PBS, ECN, VNP20009, SM-EV, SM@CMC-KP101(i.t)+NIR, and SM@CMC-KP101(i.v). Representative data from three independent experiments (n=3 mice per group) are shown.

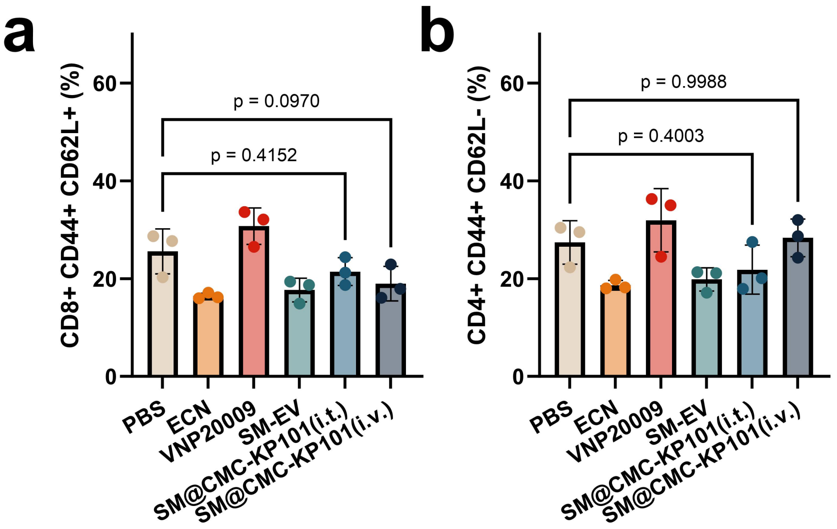

Supplementary Fig 20. Quantification of memory T Cell populations in CT26 tumor-bearing mice after intratumoral injection. (a) Percentage of CD8^{+} T cells expressing CD44^{+}CD62L^{+} (central memory phenotype) in the spleen at the end of the treatment period, following intratumoral injection with the indicated treatments. (b) Percentage of CD4^{+} T cells expressing CD44^{+}CD62L^{-} (effector memory phenotype) in the spleen at the end of the treatment period. Treatments included PBS, ECN, VNP20009, SM-EV, SM@CMC-KP101(i.t) +NIR, and SM@CMC-KP101(i.v). Data are presented as mean ± SD of n=3 biologically independent animals per group. Statistical significance compared to the PBS control group was determined by a two-sided ordinary one-way ANOVA followed by Dunnett's post-hoc test. Exact P values are indicated within the figure.

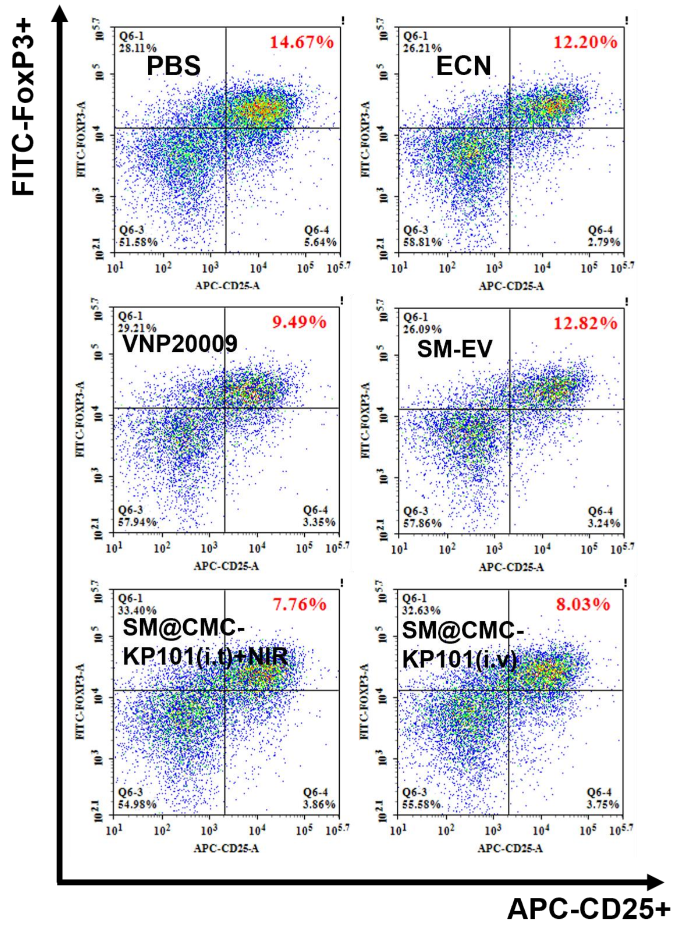

Supplementary Fig 21. Representative flow cytometry analysis of regulatory T cells (Tregs) in the spleen. The population of Tregs was identified as CD25+FoxP3+ cells within the gated CD4+ T cell population. Representative pseudo-color plots show the percentage of Tregs in the spleens of CT26 tumor-bearing mice following treatment with PBS, ECN, VNP20009, SM-EV, SM@CMC-KP101 (i.t.) + NIR, or SM@CMC-KP101 (i.v.).

50

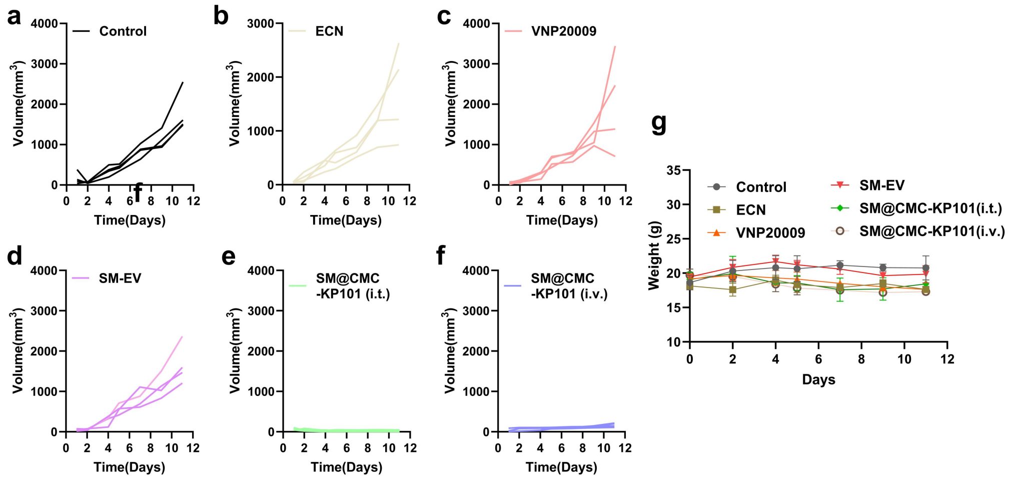

Supplementary Fig 22. (a-f) Individual tumor growth curves for mice treated with (a) Control (PBS), (b) ECN, (c) VNP20009, (d) SM-EV, (e) SM@CMC-KP101(i.t) (intratumoral injection)+NIR, and (f) SM@CMC-KP101 (i.v) (intravenous injection). Tumor volume (mm^3)

is plotted over time (days). The arrow indicates the start of treatment. (g) Mouse body weight (g) over time (days) for each treatment group. Data represent the mean ± SD of four mice per group.

51

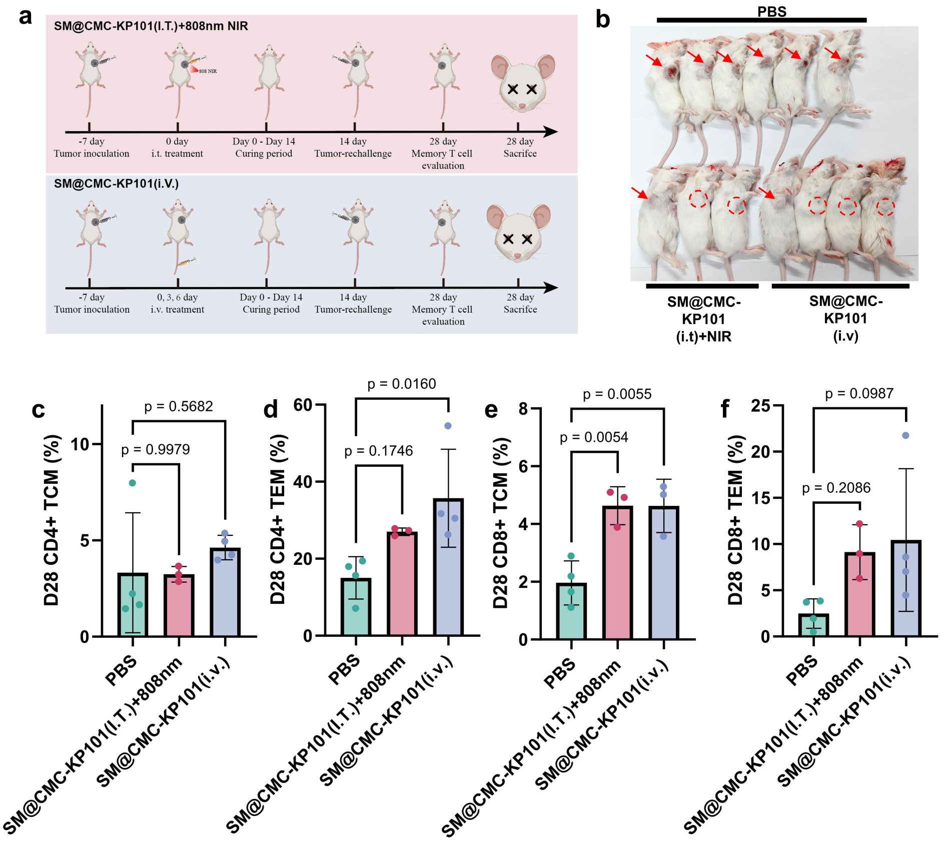

Supplementary Fig 23. Evaluation of long-term systemic immunological memory against tumor rechallenge. (a) Schematic illustration of the experimental timeline for the tumor rechallenge study. Mice cured by SM@CMC-KP101 (i.t.) + NIR or SM@CMC-KP101 (i.v.) treatments (tumor-free on day 14) were rechallenged with CT26 tumor cells in the contralateral axilla. (b) Representative photographs of mice on day 28 post-inoculation. Red arrows indicate tumor growth in the PBS control group, while red dashed circles indicate tumor-free sites in the treated groups, demonstrating protective immunity. (c-f) Flow cytometry analysis of memory T cell populations in the spleen on day 28. Quantification of the proportions of (d) CD4+ central memory T cells (TCM , $CD44^{+}CD62L^{+}$), (d) CD4+ effector memory T cells (TEM , $CD44^{+}CD62L^{-}$), (e) $CD8^{+}$ TCM , and (f) $CD8^{+}$ TEM. Data in c-f are presented as mean ± SD of n=3 biologically independent animals per group. Statistical significance compared to the PBS control group was determined by a two-sided ordinary one-way ANOVA followed by Dunnett's post-hoc test. Exact P values are indicated within the figure.

52

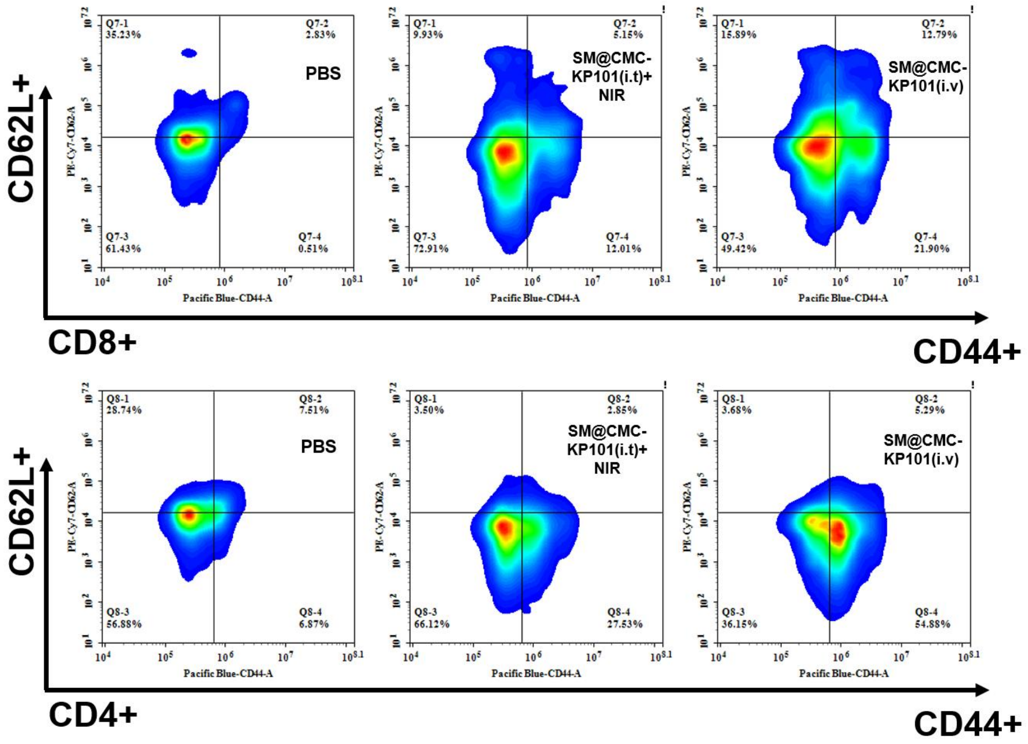

Supplementary Fig 24. Representative flow cytometry analysis of T cell memory phenotypes in the spleen of CT26 tumor-bearing mice. Flow cytometry density plots showing the expression of CD44$^+$ and CD62L$^+$ on gated (top row) CD8$^+$ T cells and (bottom row) CD4$^+$ T cells isolated from the spleen at the end of the treatment period. The plots illustrate the distribution of naïve (CD62L$^+$CD44$^-$), central memory (CD62L$^+$CD44$^+$), and effector memory (CD62L$^-$CD44$^+$) T cell subsets. Treatment groups shown include PBS, SM@CMC-KP101(i.t)+NIR, and SM@CMC-KP101(i.v.). Data are representative of three independent experiments (n=3 mice per group).

53

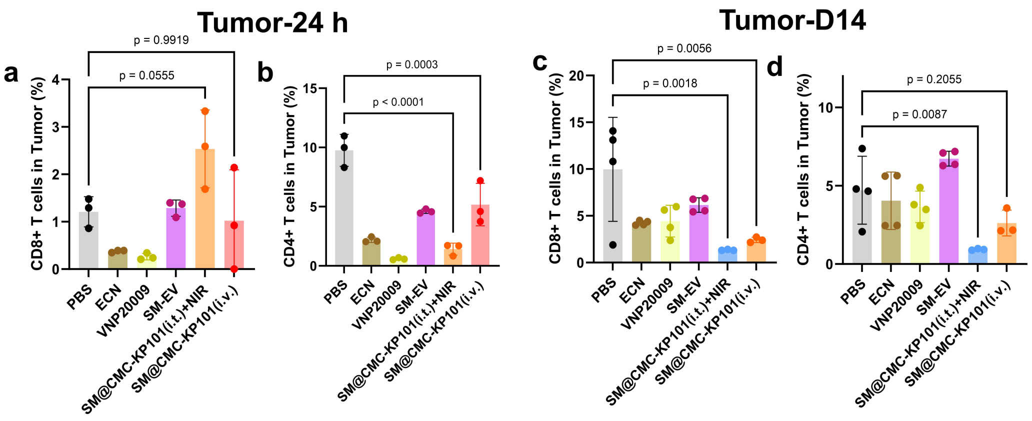

Supplementary Fig 25. Analysis of T cell infiltration in the tumor microenvironment. Flow cytometry quantification of tumor-infiltrating lymphocytes (TILs) harvested from CT26 tumor-bearing mice at (a-b) 24 hours and (c-d) 14 days post-treatment. The proportions of the following T cell subsets were analyzed across the indicated treatment groups: (a, c) CD8$^+$ cytotoxic T cells (gated as CD3$^+$CD8$^+$); (b, d) CD4+ helper T cells (gated as

CD3⁺CD4⁺). Data are presented as mean ± SD of n=3 biologically independent animals per group. Statistical significance compared to the PBS control group was determined by a two-sided ordinary one-way ANOVA followed by Dunnett's post-hoc test. Exact *P* values are indicated within the figure.

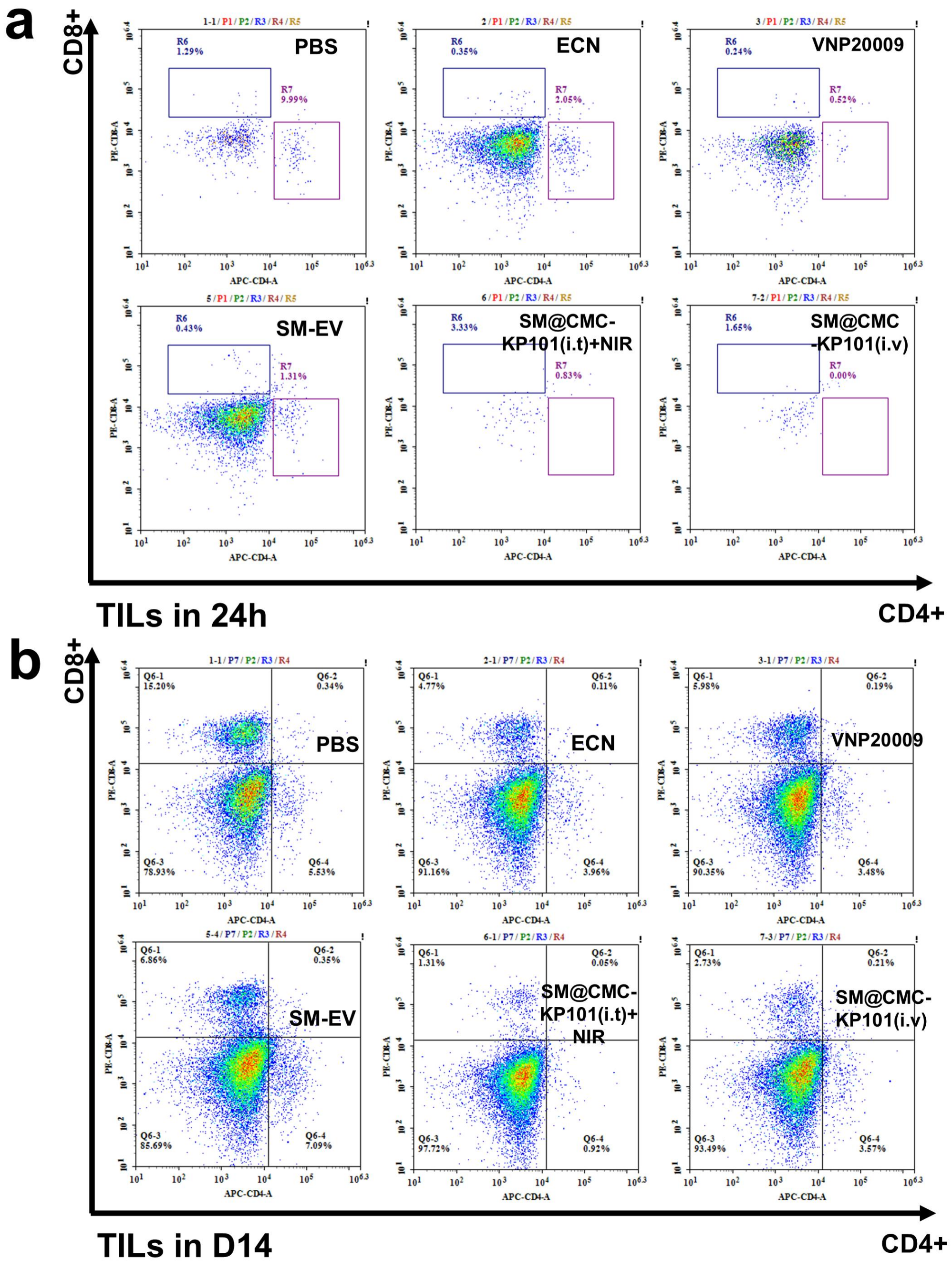

Supplementary Fig 26. Flow cytometric analysis of tumor-infiltrating lymphocytes (TILs) in CT26 tumor-bearing mice. Tumor tissues were harvested and dissociated for analysis. The dot plots display CD8⁺ (y-axis) versus CD4⁺ (x-axis) expression on cells gated as CD45⁺CD3⁺ T cells. (a) Representative plots showing TIL subpopulations at 24 hours post-treatment. (b) Representative plots showing TIL subpopulations at Day 14 post-treatment. Treatment groups include PBS, ECN, VNP20009, SM-EV,

SM@CMC-KP101(i.t)+NIR, and SM@CMC-KP101(i.v). Numbers in the gates/quadrants indicate the percentage of cells within the CD45+CD3+ parent population.

55

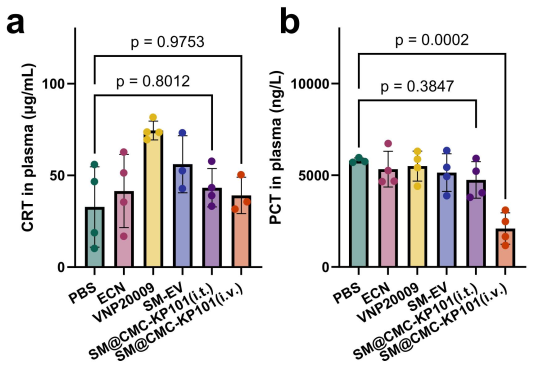

Supplementary Fig 27. Evaluation of systemic immune response and biosafety markers in plasma. Quantification of (a) calreticulin (CRT) levels and (b) procalcitonin (PCT) levels in the plasma of mice following indicated treatments. Plasma samples were collected at D11 post-treatment. Data are presented as mean ± SD of n=4biologically independent animals per group. Statistical significance compared to the PBS control group was determined by a two-sided ordinary one-way ANOVA followed by Dunnett's post-hoc test. Exact *P* values are indicated within the figure.

56

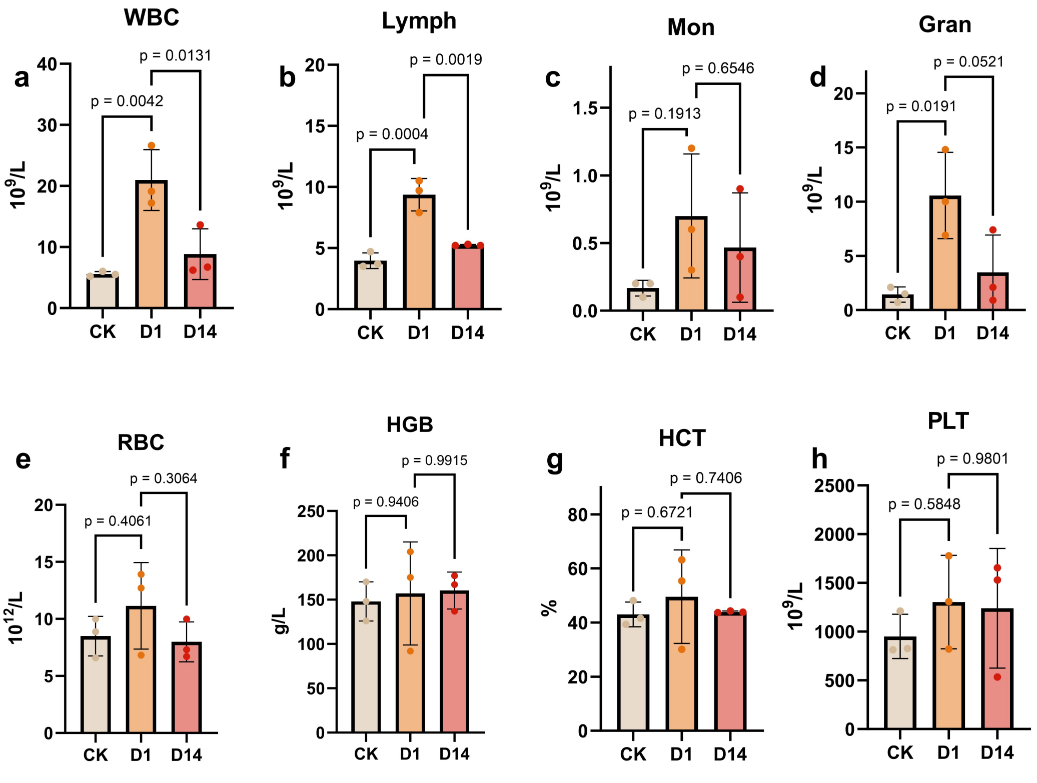

Supplementary Fig 28: Hematological analysis of mice in the SM@CMC-KP101 photothermal therapy group. Complete blood counts were performed on days 1 and 14 post-treatment to assess potential systemic toxicity. This figure displays the white blood cell count (WBC, panel a), lymphocyte count (Lymph, panel b), monocyte count (Mon, panel c),

granulocyte count (Gran, panel d), red blood cell count (RBC, panel e), hemoglobin concentration (HGB, panel f), hematocrit (HCT, panel g), and platelet count (PLT, panel h) at baseline (CK), Day 1 (D1), and Day 14 (D14). Data are presented as mean ± SD of n=3 biologically independent animals. Statistical significance compared to the baseline (CK) group was determined by a two-sided ordinary one-way ANOVA followed by Dunnett's post-hoc test. Exact *P* values are indicated within the figure.

57

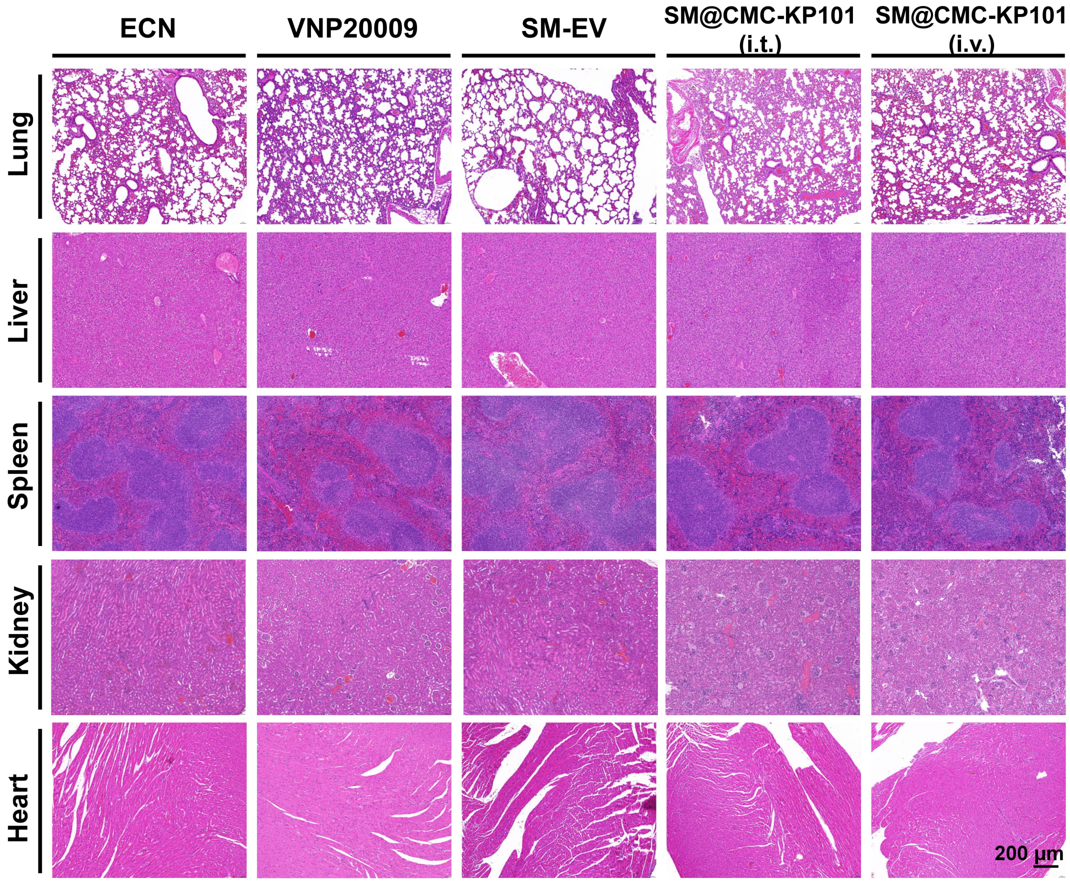

Supplementary Fig 29: Histopathological evaluation of major organs by H&E staining. Representative sections of five organs—Lung, Liver, Spleen, Kidney, and Heart (top to bottom)—from mice in different treatment groups (left to right): ECN, VNP20009, SM-EV, SM@CMC-KP101 intratumoral (i.t.), and SM@CMC KP101 intravenous (i.v.). All samples were stained with hematoxylin and eosin (H&E). Images show representative fields; scale bars are 200 μm.

58

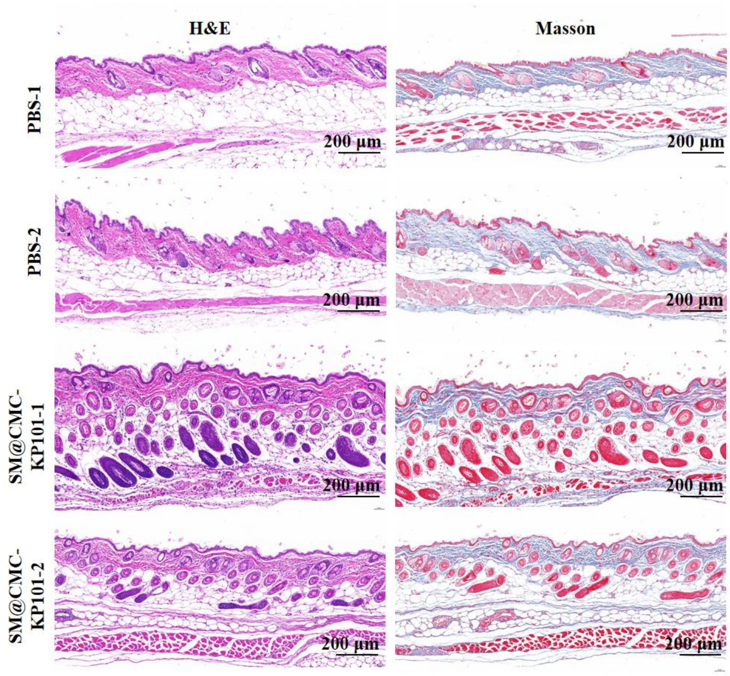

Supplementary Fig 30. Comparison of skin samples between the PBS control group and the SM@CMC-KP101 treated group. The left column shows H&E staining, and the right column shows Masson's trichrome staining. The upper and lower rows represent the PBS group and the treatment group (e.g., treated with SM@CMC-KP101), respectively. A1/A2 are from the PBS group (H&E/Masson), and B1/B2 are from the treatment group (H&E/Masson). All scale bars are 200 µm.

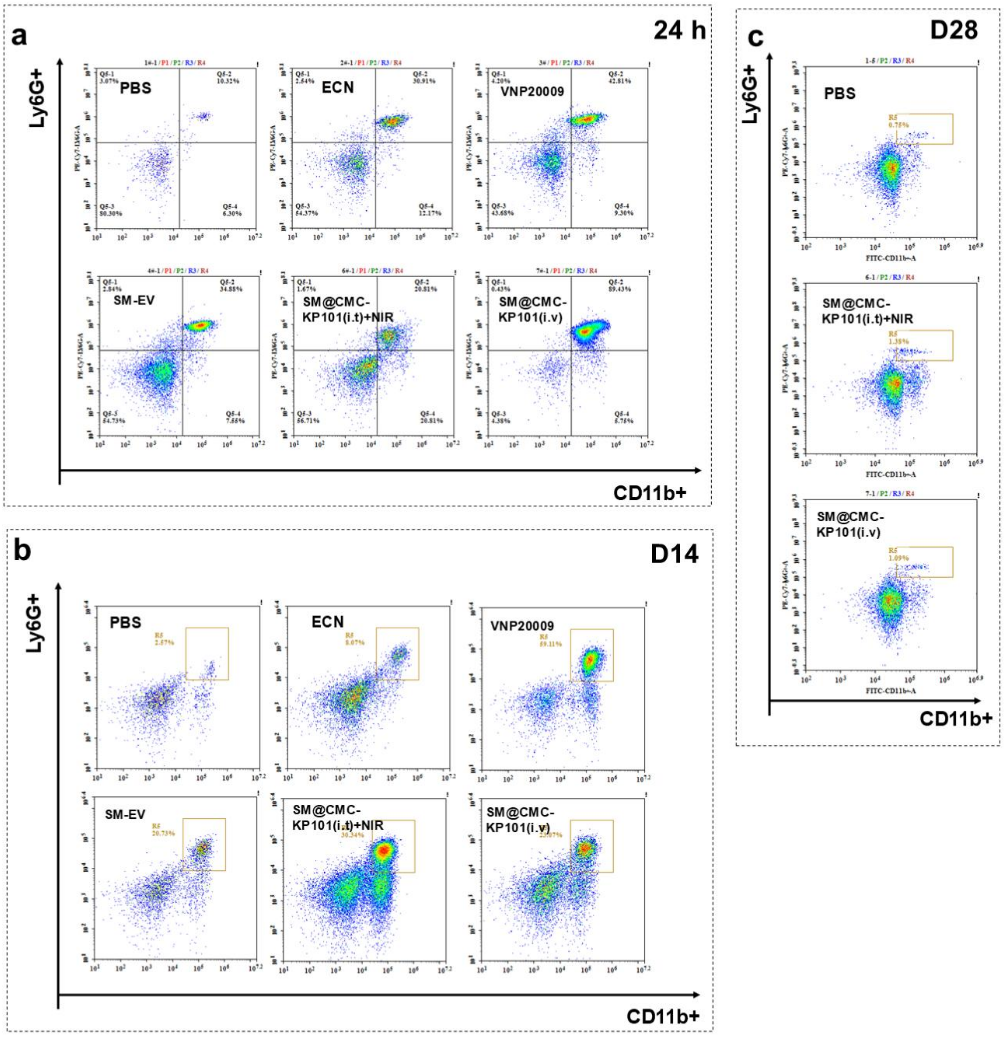

Supplementary Fig 31. Representative flow cytometry analysis of blood samples from CT26 tumor-bearing mice at 1, 14, and 28 days post-administration. Quantification of CD11b+ and Ly6G+ populations within gated CD45+ cells on (a) Day 1, (b) Day 14, and (c) Day 28 following treatment with PBS, ECN, VNP20009, SM-EV, SM@CMC-KP101 (i.t.) + NIR, or SM@CMC-KP101 (i.v.).

62

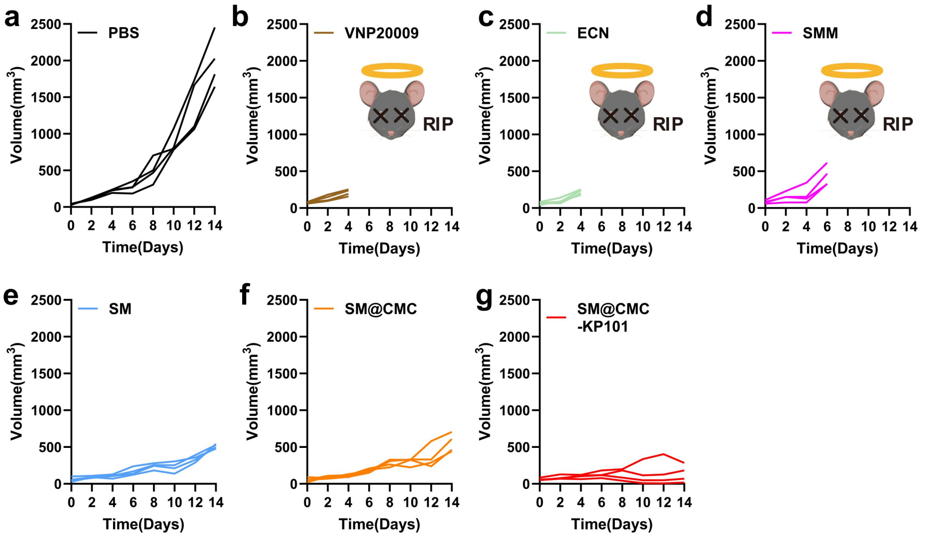

Supplementary Fig 32. Effect of Various Treatments on Tumor Growth in CT26 Tumor-Bearing Mice. (a-g) Individual tumor growth curves for mice treated with (a) PBS (control), (b) VNP20009, (c) ECN, (d) SMM, (e) SM, (f) SM@CMC, and (g) SM@CMC-KP101. Tumor volume (mm³) is plotted over time (days). Note: Panels (b-d) include "RIP" images, indicating that all mice in these treatment groups died by day 4 or day 6 due to treatment intolerance. Data represent the mean ± SD of four mice per group.

63

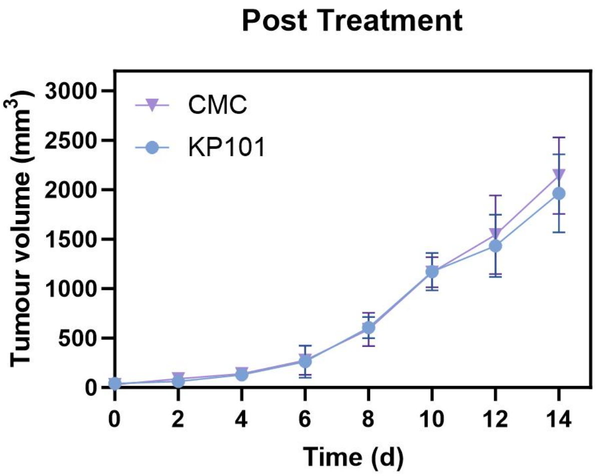

Supplementary Fig 33. Tumor growth kinetics of single-agent treatments for synergy analysis.

Time-dependent tumor volume changes in CT26-bearing mice treated with KP101 alone or CMC alone (equivalent doses to the combination therapy) via intravenous injection. Data are presented as mean ± SD (n = 5). Both single components showed limited inhibition of tumor growth, serving as the baseline for calculating the Coefficient of Drug Interaction (CDI).

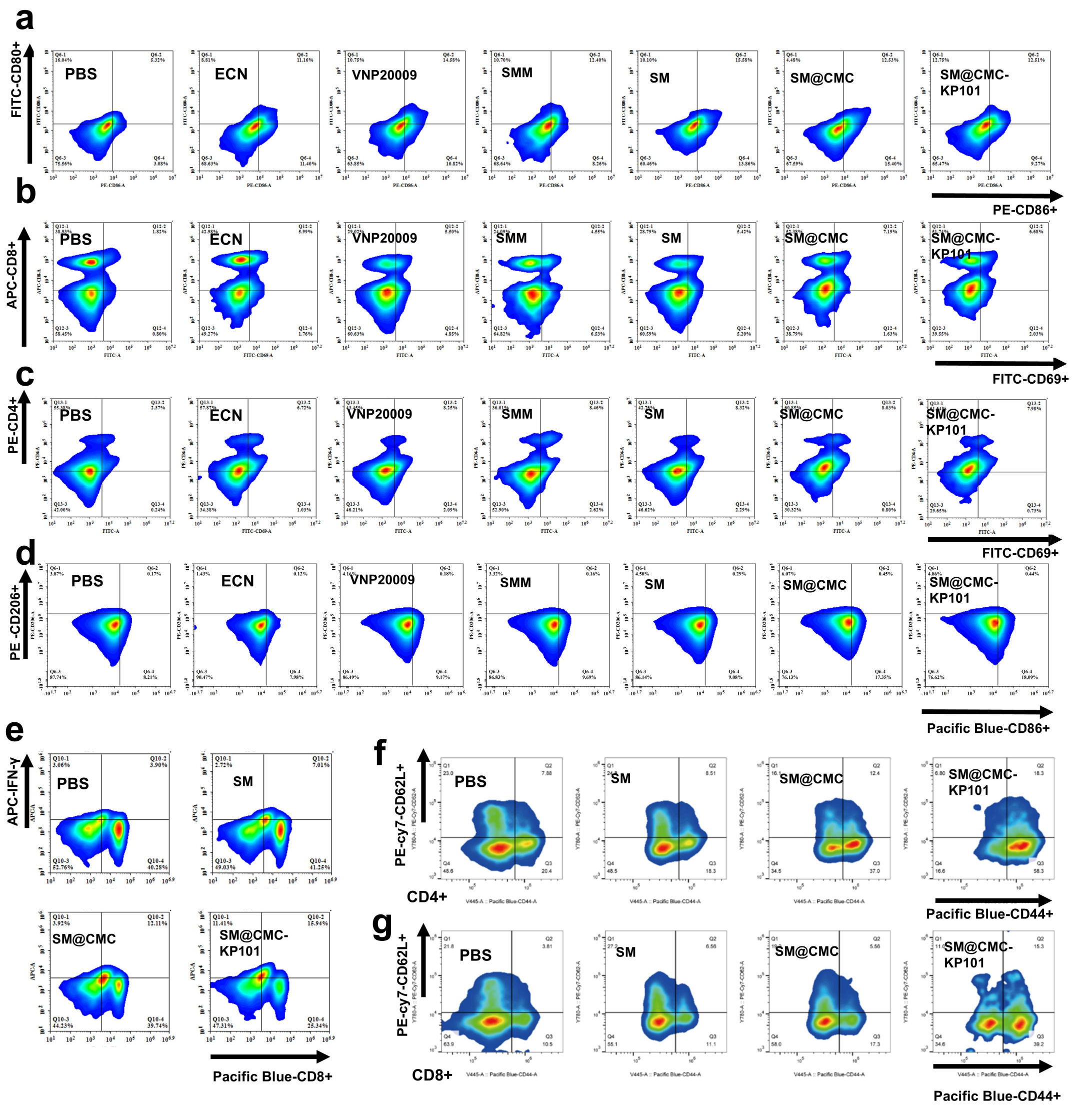

Supplementary Fig 34. Representative Flow Cytometry Analysis of B16F10 Tumor-Bearing Mice after Intravarous Injection. (a) The impact of different treatments (PBS, ECN, VNP20009, SMM, SM, SM@CMC, SM@CMC-KP101) on the expression of $CD80^{+}$ and $CD86^{+}$ on gated $CD11c^{+}$ dendritic cells (DCs) from tumor-draining lymph nodes. (b) Flow cytometry dot plots showing $CD8^{+}$ T cell populations on gated $CD69^{+}$ dendritic cells (DCs) from tumor-draining lymph nodes, 24 hours post-treatment. (c) Flow cytometry dot plots showing $CD4^{+}$ T cell populations on gated $CD69^{+}$ dendritic cells (DCs) from tumor-draining lymph nodes, 24 hours post-treatment. (d) Flow cytometry dot plots showing the expression of $CD206^{+}$ and $CD86^{+}$ on gated bone marrow-derived macrophages, 24 hours post-treatment in vitro. (e) Flow cytometry dot plots showing IFN-γ expression in gated $CD8^{+}$ T cells from the spleen at the end of the treatment period. (f) Flow cytometry dot plots showing CD44 and CD62L expression on gated $CD4^{+}$ T cells from the spleen at the end of the treatment period. (g) Flow cytometry dot plots showing CD44 and CD62L expression on gated $CD8^{+}$ T cells from the spleen at the end of the treatment period. Treatments included PBS, ECN, VNP20009, SMM, SM, SM@CMC, SM@CMC-KP101. Representative data from three independent experiments (n=3 mice per group) are shown.

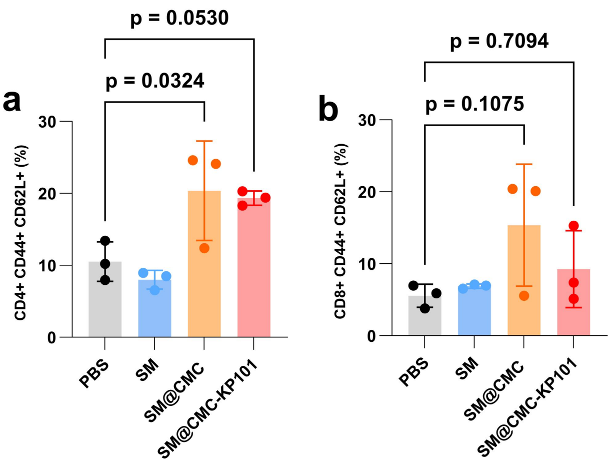

Supplementary Fig 35. Statistical percentage of central memory T cells in the spleen measured by flow cytometry. **(a)** Percentage of $CD4^{+}CD44^{+}CD62L^{+}$ T cells. **(b)** Percentage of $CD8^{+}CD44^{+}CD62L^{+}$ T cells. Treatments included PBS (control), SM, SM@CMC, and SM@CMC-KP101. Data are presented as mean ± SEM of n=3 biologically independent animals per group. Statistical significance compared to the PBS control group was determined by a two-sided ordinary one-way ANOVA followed by Dunnett's post-hoc test. Exact *P* values are indicated within the figure.

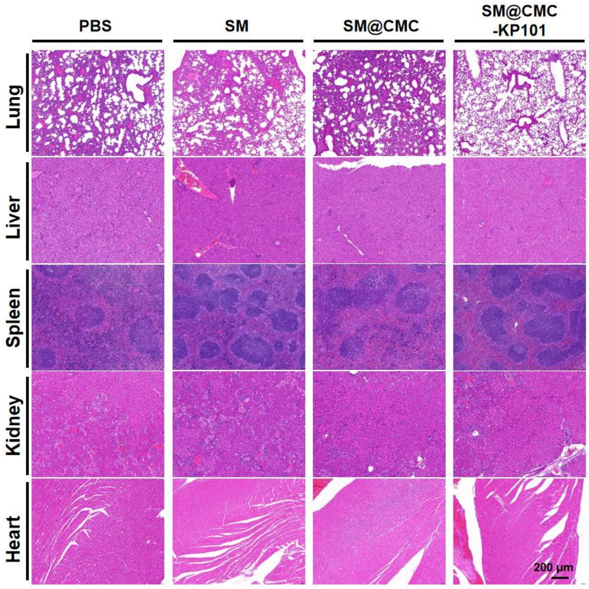

Supplementary Fig 36. Systemic biosafety evaluation after intravenous administration. Representative hematoxylin and eosin (H&E) stained images of major organs (lung, liver, spleen, kidney, and heart) from mice 14 days after a single intravenous injection of PBS, SM, SM@CMC, or SM@CMC-KP101. The bacterial formulations were administered at a dose of 1 × 10^8 CFU per mouse. No noticeable histopathological abnormalities or lesions were observed in any of the treatment groups, indicating excellent systemic tolerability at the therapeutic dose. Scale bar = 200 μm.

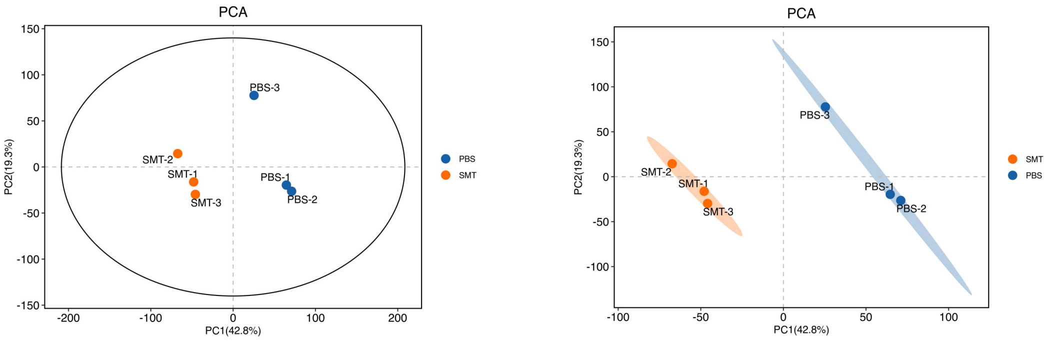

Supplementary Fig 37. Principal component analysis (PCA) based on the expression levels of verified (trusted) proteins across different samples. Each dot represents an individual sample, color - coded to indicate its respective group (orange for SMT (SM@CMC-KP101), blue for PBS). The distance between any two points reflects their overall degree of difference: samples with greater similarity cluster closer together, whereas more divergent samples appear farther apart. The PCA provides a multivariate overview of the variations in protein expression and highlights the distinct grouping tendencies of SM@CMC-KP101 versus PBS treatments.

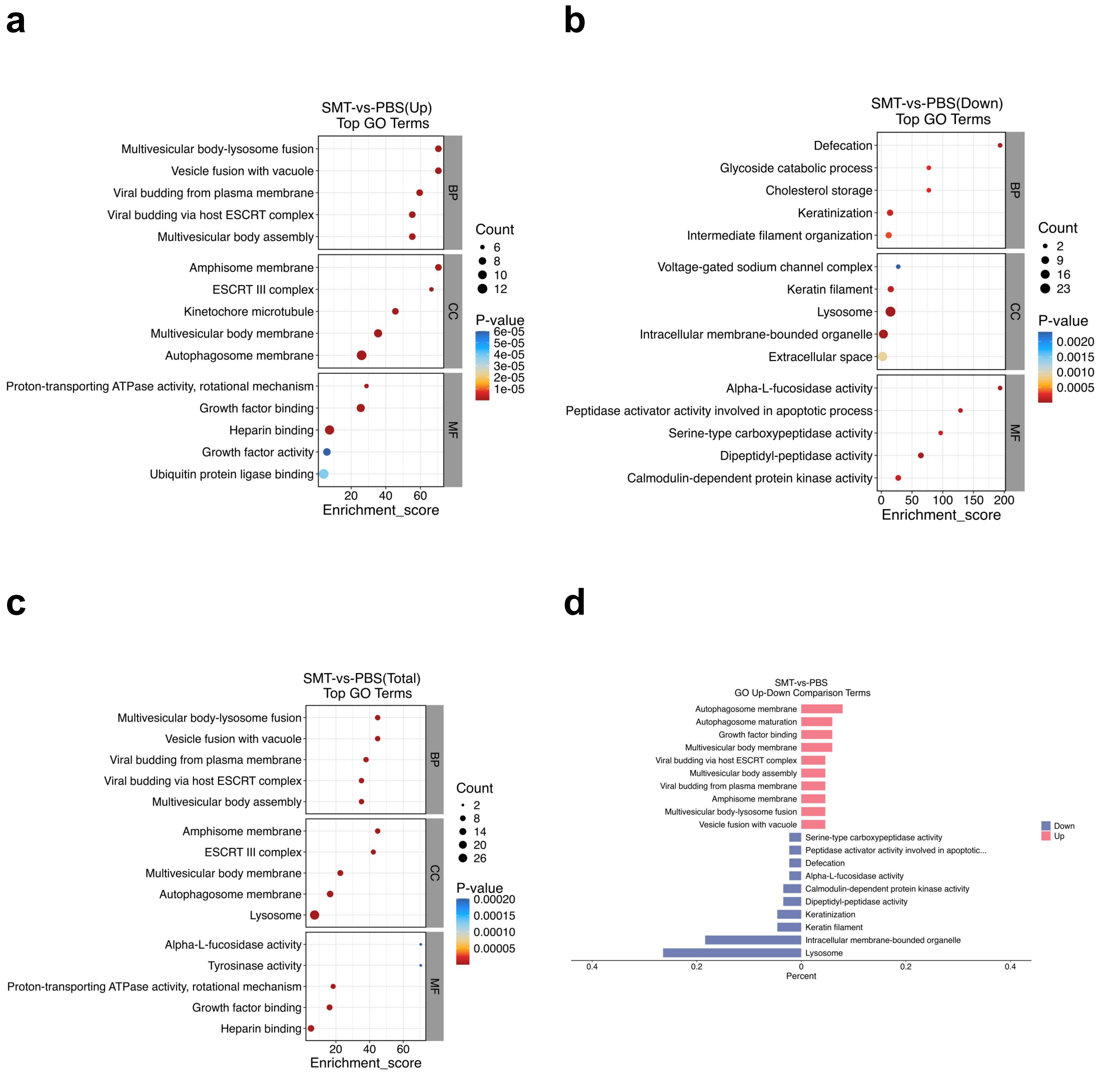

Supplementary Fig 38. GO functional enrichment analysis reveals significant GO terms of differentially expressed genes (DEGs) in SM@CMC-KP101 versus PBS, including upregulated, downregulated, and total genes. Bubble size indicates the number of genes within each term, and the color gradient (blue to red) represents statistical significance (p-value), with red indicating higher significance.

(a) GO enrichment analysis of upregulated genes in SM@CMC-KP101 versus PBS.

(b) GO enrichment analysis of downregulated genes in SM@CMC-KP101 versus PBS.

(c) GO enrichment analysis of total DEGs in SM@CMC-KP101 versus PBS.

(d) Comparative distribution of upregulated and downregulated GO terms from SM@CMC-KP101 versus PBS.

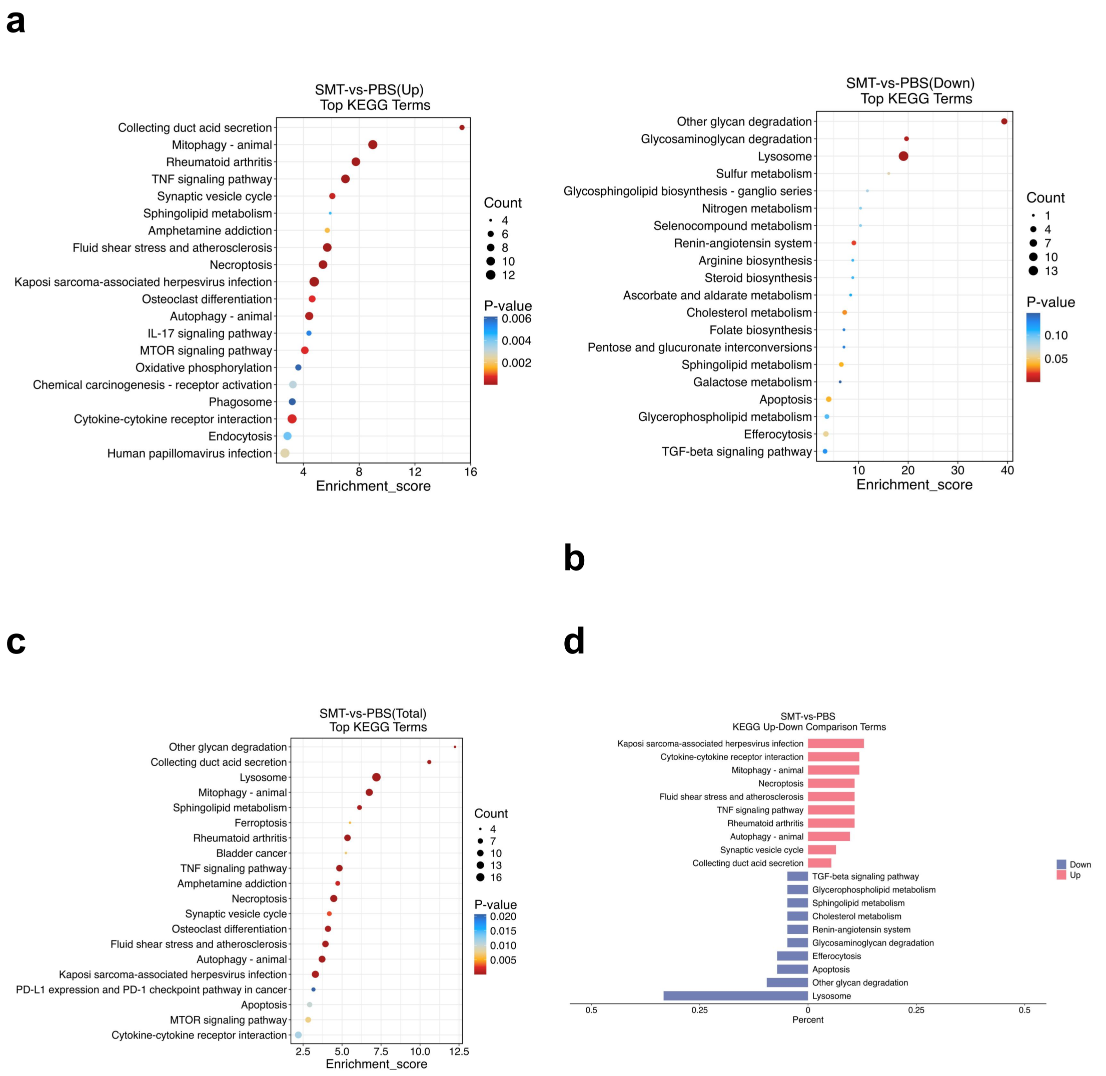

Supplementary Fig 39. KEGG functional enrichment analysis reveals significant KEGG terms of differentially expressed genes (DEGs) in SM@CMC-KP101 versus PBS, including upregulated, downregulated, and total genes. Bubble size indicates the number of genes within each term, and the color gradient (blue to red) represents statistical significance (p-value), with red indicating higher significance.

(a) KEGG enrichment analysis of upregulated genes in SM@CMC-KP101 versus PBS.

(b) KEGG enrichment analysis of downregulated genes in SM@CMC-KP101 versus PBS.

(c) KEGG enrichment analysis of total DEGs in SM@CMC-KP101 versus PBS.

(d) Comparative distribution of upregulated and downregulated KEGG terms from SM@CMC-KP101 versus PBS.

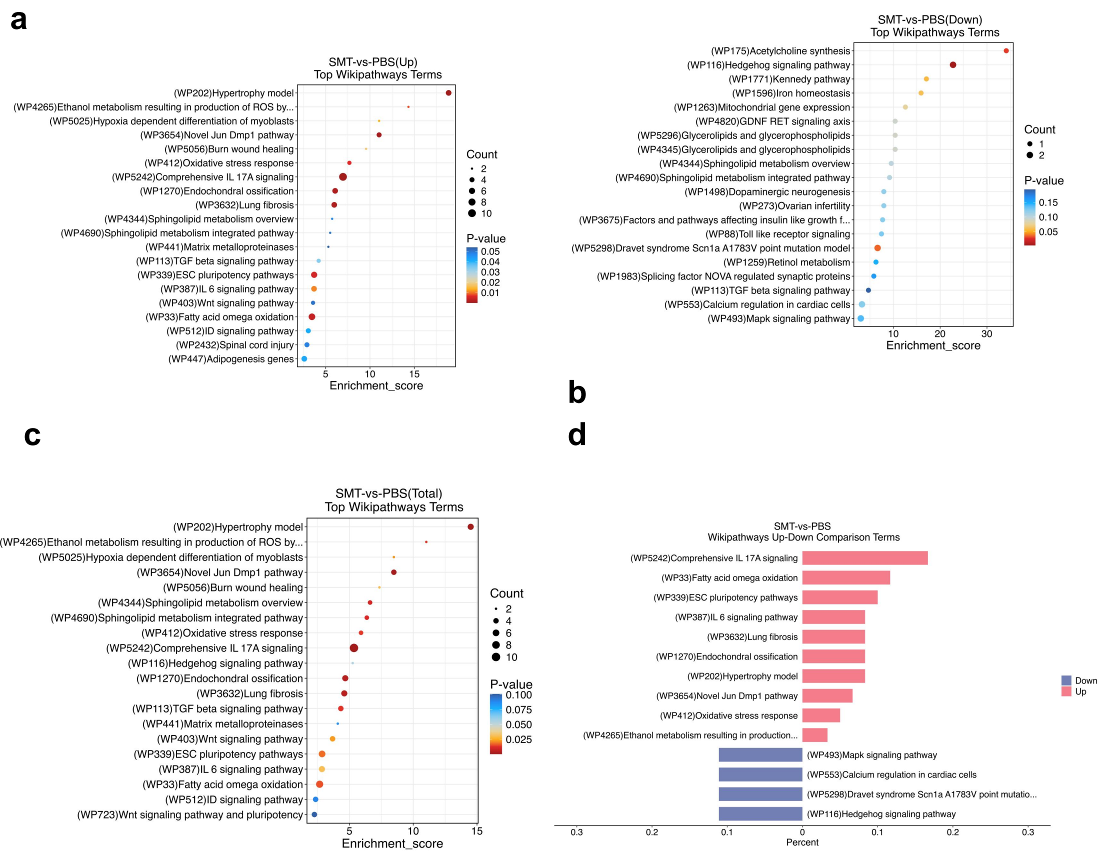

Supplementary Fig 40. Wikipathways functional enrichment analysis reveals significant Wikipathways terms of differentially expressed genes (DEGs) in SM@CMC-KP101versus PBS, including upregulated, downregulated, and total genes. Bubble size indicates the number of genes within each term, and the color gradient (blue to red) represents statistical significance (p-value), with red indicating higher significance.

(a) Wikipathways enrichment analysis of upregulated genes in SM@CMC-KP101 versus PBS.

(b) Wikipathways enrichment analysis of downregulated genes in SM@CMC-KP101 versus PBS.

(c) Wikipathways enrichment analysis of total DEGs in SM@CMC-KP101versus PBS.

(d) Comparative distribution of upregulated and downregulated Wikipathways terms from SM@CMC-KP101 versus PBS.

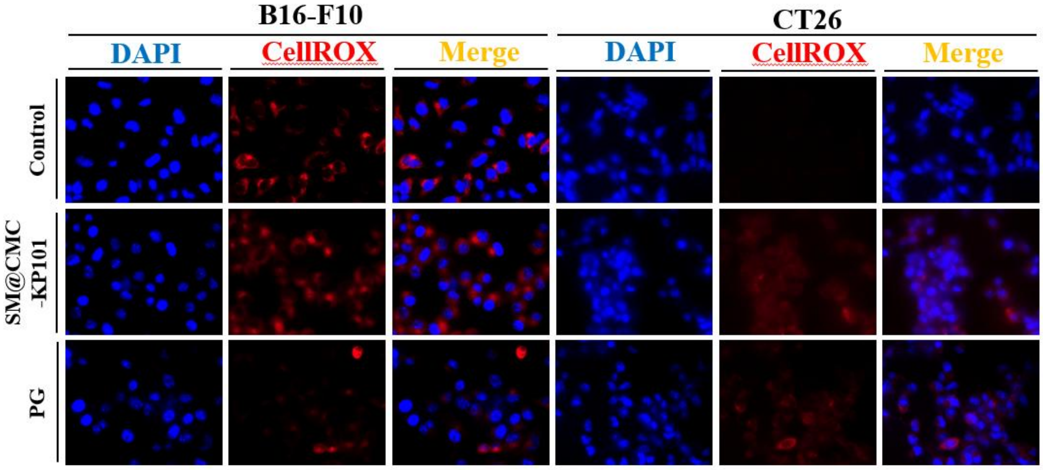

Supplementary Fig 41. Detection of reactive oxygen species (ROS) in B16-F10 and CT26 cells treated with PBS, SM@CMC-KP101 and PG, using CellROX staining.

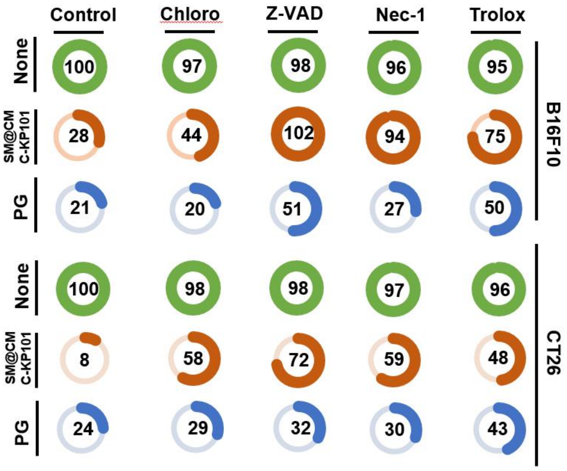

Supplementary Fig 42. The percentage of cell viability in B16-F10 and CT26 cell lines treated with PBS, SM@CMC-KP101 and PG in the presence of various inhibitors (autophagy inhibitor (Chloroquine), apoptosis inhibitor (Z-VAD-FMK), necroptosis inhibitor (Nec-1), and ROS scavenger (Trolox)). Data represent the mean ± SD of four independent experiments.

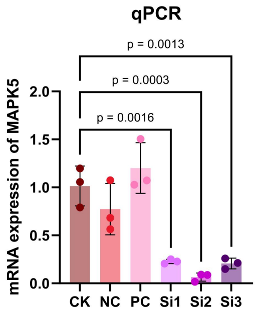

Supplementary Fig 43. qPCR analysis of MAPK5 mRNA expression in B16F10 cells after transfection with MK5 siRNAs. B16F10 cells were transfected with a negative control siRNA (NC), a positive control (PC), or one of three different MK5 siRNAs (Si1, Si2, Si3). MAPK5 mRNA expression was quantified by qPCR. Data are presented as mean ± SD of n=3 biologically independent samples. Statistical significance compared to the CK group was determined by a two-sided ordinary one-way ANOVA followed by Dunnett's post-hoc test. Exact *P* values are indicated within the figure.

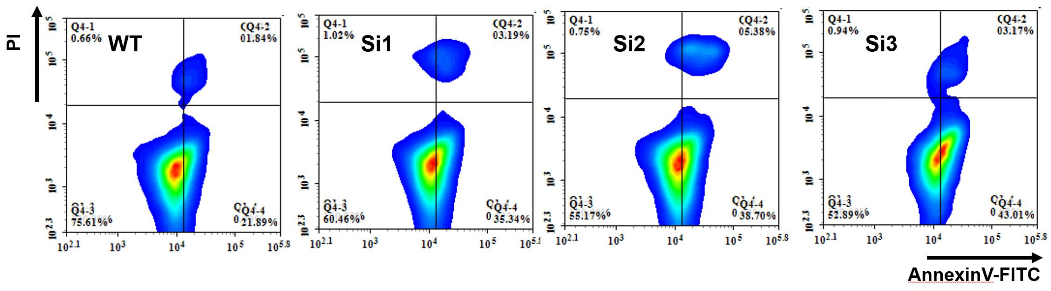

Supplementary Fig 44. Representative flow cytometry plots showing Annexin V-FITC and PI staining of B16F10 cells subjected to siRNA-mediated MAPKAPK5 gene silencing using specific siRNAs (Si1, Si2, Si3) or treated with equal volumes of transfection reagent (WT).

Supplementary Table 1 Abbreviation-to-sample mapping

Abbreviation	Full name
Control	PBS treated
ECN	*Escherichia coli* Nissle1917
JM109	*Escherichia coli* JM109
VNP20009	*Salmonella typhimurium* VNP20009
SMM	*Serratia marcescens* JC11 with pre-synthesized prodigiosin
SM	*Serratia marcescens* JC11 △ msbB with pre-synthesized prodigiosin
SM-EV	SM without PG production (empty-vector control), which can be obtained by incubating at 42℃ for 24 hours.
SM@CMC	SM coated with CMC
SM@CMC-KP101	SM@CMC linked with KP101
PG	Prodigiosin
SM@CMC-KP101(i.t.)+NIR	SM@CMC-KP101 administered intratumorally (i.t.), followed by 808 nm NIR irradiation for 3 min.
SM@CMC-KP101(i.v.)	SM@CMC-KP101 administered intravenously (i.v.).

Supplementary Table 2

Microorganism	Strategies	Prodigiosin yield	Reference
Serratia marcescens	Microwave mutagenesis	6500 mg/L	[1]
S. marcescens JNB5 - 1	de novo polynucleotide fragments (PNFs) and the introduction of disulfide bonds to O-methyl transferase (PigF) and oxidoreductase (PigN)	8650 mg/L	[2]
S. marcescens JNB5 - 1	Disruption of dacA	227.94 mg/L	[3]
S. marcescens JNB5 - 1	Disruption of CpxR, integrate a fusion of proC, serC, and metH	5830 mg/L	[4]
S. marcescens JNB5 - 1	Promoter engineering for overexpressing of prodigiosin synthesis activator OmpR and PsrA	10250 mg/L	[5]
P. putida KT2440	Integrated pig cluster of *S. marcescens* ATCC 274 to P. putida KT2440 through λRed/Cas9 recombination	1100 mg/L	[6]

Supplementary Table 3 Detailed information of FACS antibody

Supplier	Item number	Antibody
eBioscience™	65-0865-14	FIXABLE VIABILITY DYE EF780
BD	752417	BV480 Rat Anti-Mouse CD45
BD	557396	FITC Rat Anti-CD11b(M1/70)
eBioscience™	12-2061-82	CD206 (MMR) Monoclonal Antibody (MR6F3), PE
BD	553051	APC Rat Anti-Mouse CD4(RM4-5)
BD	553032	Ms CD8a PE 53-6.7 100ug
BD	563970	Ms CD44 BV421 IM7 50ug
BD	560516	PE-Cy7 Rat Anti-Mouse CD62L(MEL-14)
absin	abs9477-200T	Fc Receptor Blocking Solution, Mouse
BioLegend	117310	APC anti-mouse CD11c
BioLegend	104705	FITC anti-mouse CD80
BioLegend	105022	Pacific Blue™ anti-mouse CD86
BioLegend	100204	FITC anti-mouse CD3
BioLegend	104505	antimouse CD69-FITC
BioLegend	100512	PE anti-mouse CD4
BioLegend	100725	Pacific Blue™ anti-mouse CD8a
BioLegend	505810	APC anti-mouse IFN-γ
Introvigen	#11-5773-82	FITC anti-mouse FOXP3
Introvigen	#17-0251-81	antimouse CD25-APC

Supplementary Table 4 Detailed information of WB antibody

Antibody	Supplier	Item number	ratio
Pink1	Abmart	PK05715S	1:1000
Cathepsin D (CTSB)	Beyotime	AF1645	1:1000
β-Actin	Beyotime	AF2815	1:1000
LC3B	Beyotime	AB2023	1:1000
caspase3	Abclonal	A19664	1:1000
cleaved-caspase3	Abmart	TA7022	1: 500
Phospho-MLKL(Ser358)	cellsignal	#37333	1:1000
MLKL	cellsignal	#37705	1:1000

Supplementary Table 5

primer	sequence
msbB-Up-F	caatttgtggaatcccgggagagctCTGAGGTGGTGATCGCCAAACGCAG
msbB-Up-R	ACCGGCGTTAGCGGTAGAAAGGAGCACGTGCTCTTCGCTTAGCGATTC
msbB-Down-F	CGCTAAGCGAAGAGCACGTGCTCCTTTCTACCGCTAACG
msbB-Down-R	cgataccgtcgaccctcgaGCTGGCCGTTTTGCCTCTGGCGATAC

Supplementary Table 6

Item Name	Oligo Sequence
Mapkapk5_si1_S	CCAAAGGAUAUGCGGAAAAdTdT
Mapkapk5_si1_AS	UUUUCCGCAUAUCCUUUGGdTdT
Mapkapk5_si2_S	GAUUCACAGAUAAAGUUGAdTdT
Mapkapk5_si2_AS	UCAACUUUAUCUGUGAAUCdTdT
Mapkapk5_si3_S	CUUGAUCGUCCAAAAGCUAdTdT
Mapkapk5_si3_AS	UAGCUUUUGGACGAUCAAGdTdT

TableS7

primer	sequence
CD206-F	CTCTGTTCAGCTATTGGACGC
CD206-R	CGGAATTTCTGGGATTCAGCTTC
IL10-F	GCTCTTACTGACTGGCATGAG
IL10-R	CGCAGCTCTAGGAGCATGTG
TNFa-F	CCCTCACACTCAGATCATCTTCT
TNFa-R	GCTACGACGTGGGCTACAG
iNOS-F	CACCTTGGAGTTCACCCAGT
iNOS-R	ACCACTCGTACTTGGGATGC
β-actin-F	AGCCATGTACGTAGCCATCC
β-actin-R	CTCTCAGCTGTGGTGGTGAA

Supplementary References

1. Liu, X. et al. Mutant breeding of Serratia marcescens strain for enhancing prodigiosin production and application to textiles. *Prep Biochem Biotechnol* **43**, 271-284 (2013). doi: 10.1080/10826068.2012.721850.
2. Sun, Y. et al. Enhanced Prodigiosin Production in Serratia marcescens JNB5-1 by Introduction of a Polynucleotide Fragment into the pigN 3' Untranslated Region and Disulfide Bonds into O-Methyl Transferase (PigF). *Appl Environ Microbiol* **87**, e0054321 (2021). doi: 10.1128/AEM.00543-21.
3. Pan, X. et al. Loss of Serine-Type D-Ala-D-Ala Carboxypeptidase DacA Enhances Prodigiosin Production in Serratia marcescens. *Front Bioeng Biotechnol* **7**, 367 (2019). doi: 10.3389/fbioe.2019.00367.
4. Sun, Y. et al. Improved Prodigiosin Production by Relieving CpxR Temperature-Sensitive Inhibition. *Frontiers in Bioengineering and Biotechnology* **8** (2020). doi: 10.3389/fbioe.2020.00344.
5. Pan, X. et al. Improving prodigiosin production by transcription factor engineering and promoter engineering in Serratia marcescens. *Front Microbiol* **13**, 977337 (2022). doi: 10.3389/fmicb.2022.977337.
6. Dauenhauer, S.A., Hull, R.A. & Williams, R.P. Cloning and expression in Escherichia coli of Serratia marcescens genes encoding prodigiosin biosynthesis. *Journal of Bacteriology* **158**, 1128-1132 (1984). doi: doi:10.1128/jb.158.3.1128-1132.1984.
