# Supplementary material for: A live biohybrid bacterial therapy based on engineered Serratia marcescens
Source: Nat Commun. 2026 Apr 7;17:4956. doi: 10.1038/s41467-026-70949-4 (PMC13234118; doi:10.1038/s41467-026-70949-4)
Supplement: Supplementary file 2 — Description of Additional Supplementary Information [file 41467_2026_70949_MOESM2_ESM.pdf]

Description of Additional Supplementary Files

File Name: Supplementary Data 1
Description: Differentially expressed proteins from DIA proteomic analysis.
