# Supplementary material for: A live biohybrid bacterial therapy based on engineered Serratia marcescens
Source: Nat Commun. 2026 Apr 7;17:4956. doi: 10.1038/s41467-026-70949-4 (PMC13234118; doi:10.1038/s41467-026-70949-4)
Supplement: Supplementary file 4 — Reporting Summary [file 41467_2026_70949_MOESM4_ESM.pdf]

nature portfolio

Corresponding author(s): weiwei

Last updated by author(s): Mar 3, 2026

Reporting Summary

Nature Portfolio wishes to improve the reproducibility of the work that we publish. This form provides structure for consistency and transparency in reporting. For further information on Nature Portfolio policies, see our Editorial Policies and the Editorial Policy Checklist.

Statistics

For all statistical analyses, confirm that the following items are present in the figure legend, table legend, main text, or Methods section.

n/a	Confirmed	
☐	☒	The exact sample size (*n*) for each experimental group/condition, given as a discrete number and unit of measurement
☐	☒	A statement on whether measurements were taken from distinct samples or whether the same sample was measured repeatedly
☐	☒	The statistical test(s) used AND whether they are one- or two-sided<br>*Only common tests should be described solely by name; describe more complex techniques in the Methods section.*
☒	☐	A description of all covariates tested
☒	☐	A description of any assumptions or corrections, such as tests of normality and adjustment for multiple comparisons
☐	☒	A full description of the statistical parameters including central tendency (e.g. means) or other basic estimates (e.g. regression coefficient) AND variation (e.g. standard deviation) or associated estimates of uncertainty (e.g. confidence intervals)
☐	☒	For null hypothesis testing, the test statistic (e.g. *F*, *t*, *r*) with confidence intervals, effect sizes, degrees of freedom and *P* value noted<br>*Give P values as exact values whenever suitable.*
☒	☐	For Bayesian analysis, information on the choice of priors and Markov chain Monte Carlo settings
☒	☐	For hierarchical and complex designs, identification of the appropriate level for tests and full reporting of outcomes
☒	☐	Estimates of effect sizes (e.g. Cohen's *d*, Pearson's *r*), indicating how they were calculated

Our web collection on statistics for biologists contains articles on many of the points above.

Software and code

Policy information about availability of computer code

Data collection	Images were acquired using a Zeiss LSM880 confocal microscope controlled by Zeiss ZEN Black software (version 3.1). Flow cytometry data were collected using NovoExpress (version 1.6.2).
Data analysis	Flow cytometry data were analyzed using FlowJo (version 10.8.1) and NovoExpress (version 1.6.2). Statistical analyses and data visualization were performed using GraphPad Prism (version 10.1.2) and Origin 2023b. Image processing and analysis were conducted using ImageJ (version 2.14.0/1.54p). Final figures were assembled using Adobe Illustrator2024, with specific schematic elements (cell and mouse icons) created using BioRender.com.

For manuscripts utilizing custom algorithms or software that are central to the research but not yet described in published literature, software must be made available to editors and reviewers. We strongly encourage code deposition in a community repository (e.g. GitHub). See the Nature Portfolio guidelines for submitting code & software for further information.

Data

Policy information about availability of data

All manuscripts must include a data availability statement. This statement should provide the following information, where applicable:

- Accession codes, unique identifiers, or web links for publicly available datasets
- A description of any restrictions on data availability
- For clinical datasets or third party data, please ensure that the statement adheres to our policy

ll data supporting the findings of this study are available within the paper and its supplementary information. Raw data generated for the figures are provided in the

source data file, specifically for Figures 1b, 1c, 1d, 1f,1 h, 2c, 2d, 2f, 2g, 2h, 3c, 3d, 3e, 3f-i, 4c, 4e-l, 4n, 4o, 4q-s, 5b, 5c, 5e, 5f-n, 6c, 6f, 6g, and Supplementary Figures 2,3,4,5,6,7,9,11,15,16,17,23,24,26,28,29,32,33,35,42,43

Research involving human participants, their data, or biological material

Policy information about studies with human participants or human data. See also policy information about sex, gender (identity/presentation), and sexual orientation and race, ethnicity and racism.

Reporting on sex and gender	Not applicable
Reporting on race, ethnicity, or other socially relevant groupings	Not applicable
Population characteristics	Not applicable
Recruitment	Not applicable
Ethics oversight	Not applicable

Note that full information on the approval of the study protocol must also be provided in the manuscript.

Field-specific reporting

Please select the one below that is the best fit for your research. If you are not sure, read the appropriate sections before making your selection.

☒ Life sciences ☐ Behavioural & social sciences ☐ Ecological, evolutionary & environmental sciences

For a reference copy of the document with all sections, see nature.com/documents/nr-reporting-summary-flat.pdf

Life sciences study design

All studies must disclose on these points even when the disclosure is negative.

Sample size	In all in vitro experiments, a minimum of three randomly selected samples were subjected to statistical analysis. Likewise, a minimum of three randomly selected samples were analyzed per in vivo experiment. Sample sizes for all experiments were determined based on effect sizes and distribution overlap documented in previous studies (Nature, 2024, 635, 453–461; Cell Metabolism, 2025, 37, 1277-1293). Although formal sample size calculations were not conducted, sample sizes were selected to provide sufficient statistical power, taking into account previous literature, experimental design, and practical resource considerations. Sample size is explicitly stated for each experimental group for individual experiments in figurecaptions and data descriptions.
Data exclusions	No data was excluded from the analysis.
Replication	Results shown in the manuscript are representative of at least three independent experiments. All our attempts at replication were successful with similar results.
Randomization	Samples and organisms were randomly allocated to experimental groups using simple randomization.
Blinding	In all experiments, investigators were blinded to group allocation during data collection and processing.

Behavioural & social sciences study design

All studies must disclose on these points even when the disclosure is negative.

Study description	*Briefly describe the study type including whether data are quantitative, qualitative, or mixed-methods (e.g. qualitative cross-sectional, quantitative experimental, mixed-methods case study).*
Research sample	*State the research sample (e.g. Harvard university undergraduates, villagers in rural India) and provide relevant demographic information (e.g. age, sex) and indicate whether the sample is representative. Provide a rationale for the study sample chosen. For studies involving existing datasets, please describe the dataset and source.*
Sampling strategy	*Describe the sampling procedure (e.g. random, snowball, stratified, convenience). Describe the statistical methods that were used to predetermine sample size OR if no sample-size calculation was performed, describe how sample sizes were chosen and provide a rationale for why these sample sizes are sufficient. For qualitative data, please indicate whether data saturation was considered, and what criteria were used to decide that no further sampling was needed.*
Data collection	*Provide details about the data collection procedure, including the instruments or devices used to record the data (e.g. pen and paper, computer, eye tracker, video or audio equipment) whether anyone was present besides the participant(s) and the researcher, and whether the researcher was blind to experimental condition and/or the study hypothesis during data collection.*

Timing	*Indicate the start and stop dates of data collection. If there is a gap between collection periods, state the dates for each sample cohort.*
Data exclusions	*If no data were excluded from the analyses, state so OR if data were excluded, provide the exact number of exclusions and the rationale behind them, indicating whether exclusion criteria were pre-established.*
Non-participation	*State how many participants dropped out/declined participation and the reason(s) given OR provide response rate OR state that no participants dropped out/declined participation.*
Randomization	*If participants were not allocated into experimental groups, state so OR describe how participants were allocated to groups, and if allocation was not random, describe how covariates were controlled.*

Ecological, evolutionary & environmental sciences study design

All studies must disclose on these points even when the disclosure is negative.

Study description	*Briefly describe the study. For quantitative data include treatment factors and interactions, design structure (e.g. factorial, nested, hierarchical), nature and number of experimental units and replicates.*
Research sample	*Describe the research sample (e.g. a group of tagged Passer domesticus, all Stenocereus thurberi within Organ Pipe Cactus National Monument), and provide a rationale for the sample choice. When relevant, describe the organism taxa, source, sex, age range and any manipulations. State what population the sample is meant to represent when applicable. For studies involving existing datasets, describe the data and its source.*
Sampling strategy	*Note the sampling procedure. Describe the statistical methods that were used to predetermine sample size OR if no sample-size calculation was performed, describe how sample sizes were chosen and provide a rationale for why these sample sizes are sufficient.*
Data collection	*Describe the data collection procedure, including who recorded the data and how.*
Timing and spatial scale	*Indicate the start and stop dates of data collection, noting the frequency and periodicity of sampling and providing a rationale for these choices. If there is a gap between collection periods, state the dates for each sample cohort. Specify the spatial scale from which the data are taken*
Data exclusions	*If no data were excluded from the analyses, state so OR if data were excluded, describe the exclusions and the rationale behind them, indicating whether exclusion criteria were pre-established.*
Reproducibility	*Describe the measures taken to verify the reproducibility of experimental findings. For each experiment, note whether any attempts to repeat the experiment failed OR state that all attempts to repeat the experiment were successful.*
Randomization	*Describe how samples/organisms/participants were allocated into groups. If allocation was not random, describe how covariates were controlled. If this is not relevant to your study, explain why.*
Blinding	*Describe the extent of blinding used during data acquisition and analysis. If blinding was not possible, describe why OR explain why blinding was not relevant to your study.*

Did the study involve field work? ☐ Yes ☐ No

Field work, collection and transport

Field conditions	*Describe the study conditions for field work, providing relevant parameters (e.g. temperature, rainfall).*
Location	*State the location of the sampling or experiment, providing relevant parameters (e.g. latitude and longitude, elevation, water depth).*
Access & import/export	*Describe the efforts you have made to access habitats and to collect and import/export your samples in a responsible manner and in compliance with local, national and international laws, noting any permits that were obtained (give the name of the issuing authority, the date of issue, and any identifying information).*
Disturbance	*Describe any disturbance caused by the study and how it was minimized.*

Reporting for specific materials, systems and methods

We require information from authors about some types of materials, experimental systems and methods used in many studies. Here, indicate whether each material, system or method listed is relevant to your study. If you are not sure if a list item applies to your research, read the appropriate section before selecting a response.

Materials & experimental systems

n/a	Involved in the study
	☒ Antibodies
	☒ Eukaryotic cell lines
☒	☐ Palaeontology and archaeology
	☒ Animals and other organisms
☒	☐ Clinical data
☒	☐ Dual use research of concern
☒	☐ Plants

Methods

n/a	Involved in the study
☒	☐ ChIP-seq
	☒ Flow cytometry
☒	☐ MRI-based neuroimaging

Antibodies

Antibodies used

For Western blot analysis, the following primary antibodies were used: Pink1 (Abmart, PK05715S, 1:1000), Cathepsin D (Beyotime, AF1645, 1:1000), β-Actin (Beyotime, AF2815, 1:1000), LC3B (Beyotime, AB2023, 1:1000), Hsp27 (Beyotime, AF0183, 1:1000), p-Hsp27(S82) (Cell Signaling Technology, 9709, 1:1000), caspase3 (Abclonal, A19664, 1:1000), cleaved-caspase3 (Abmart, TA7022, 1:500), Phospho-MLKL(Ser358) (Cell Signaling Technology, #37333, 1:1000), and MLKL (Cell Signaling Technology, #37705, 1:1000).

For flow cytometry analysis, the following antibodies were used: FIXABLE VIABILITY DYE EF780 (eBioscience, 65-0865-14), BV480 Rat Anti-Mouse CD45 (BD Biosciences, 752417), FITC Rat Anti-CD11b(M1/70) (BD Biosciences, 557396), CD206 (MMR) Monoclonal Antibody (MR6F3), PE (eBioscience, 12-2061-82), APC Rat Anti-Mouse CD4(RM4-5) (BD Biosciences, 553051), Ms CD8a PE 53-6.7 100ug (BD Biosciences, 553032), Ms CD44 BV421 IM7 50ug (BD Biosciences, 563970), PE-Cy7 Rat Anti-Mouse CD62L(MEL-14) (BD Biosciences, 560516), Fc Receptor Blocking Solution, Mouse (absin, abs9477-200T), APC anti-mouse CD11c (BioLegend, 117310), FITC anti-mouse CD80 (BioLegend, 104705), Pacific Blue™ anti-mouse CD86 (BioLegend, 105022), Pacific Blue™ anti-mouse CD80 Antibody (BioLegend, 104723), PE anti-mouse CD86 Antibody (BioLegend, 159203), FITC anti-mouse CD3 (BioLegend, 100204), anti-mouse CD69-FITC (BioLegend, 104505), PE anti-mouse CD4 (BioLegend, 100512), Pacific Blue™ anti-mouse CD8a (BioLegend, 100725), APC anti-mouse IFN-γ (BioLegend, 505810), FITC anti-mouse FOXP3 (Invitrogen, #11-5773-82), and anti-mouse CD25-APC (Invitrogen, #17-0251-81). All antibodies were diluted to the working concentration of 0.2 ug per test.

Validation

Western Blot Antibodies:
Pink1: Abmart, PK05715S, 1:1000, https://www.ab-mart.com/Goods/view?id=216281
Cathepsin D: Beyotime, AF1645, 1:1000, https://www.beyotime.com/product/AF1645.htm
β-Actin: Beyotime, AF2815, 1:1000, https://www.beyotime.com/product/AF2815.htm
LC3B: Beyotime, AB2023, 1:1000, https://www.beyotime.com/product/AB2023.htm
Hsp27: Beyotime, AF0183, 1:1000, https://www.beyotime.com/product/AF0183.htm
p-Hsp27(S82): Cell Signaling Technology, 9709, 1:1000, https://www.cellsignal.com/products/primary-antibodies/phospho-hsp27-ser82-antibody/9709
p38: Beyotime, AF1111, 1:1000, https://www.beyotime.com/product/AF1111.htm
p-p38: Cell Signaling Technology, 4511T, 1:1000, https://www.cellsignal.com/products/primary-antibodies/phospho-p38-mapk-thr180-tyr182-antibody/4511
caspase3: Abclonal, A19664, 1:1000, https://www.abclonal.com/p/caspase3-antibody-a19664
cleaved-caspase3: Abmart, TA7022, 1:500, https://www.ab-mart.com/Goods/view?id=203253
Phospho-MLKL(Ser358): Cell Signaling Technology, #37333, 1:1000, https://www.cellsignal.com/products/primary-antibodies/phospho-mlkl-ser358-d6h3n-rabbit-mab/37333
MLKL: Cell Signaling Technology, #37705, 1:1000, https://www.cellsignal.com/products/primary-antibodies/mlkl-d3g9-rabbit-mab/37705
RIPK3: Abclonal, A5431, 1:1000, https://www.abclonal.com/p/ripk3-antibody-a5431
p-RIPK3: Abclonal, AP1260, 1:1000, https://www.abclonal.com/p/phospho-ripk3-thr231-antibody-ap1260
FACS Antibodies:
FIXABLE VIABILITY DYE EF780: eBioscience, 65-0865-14, https://www.thermofisher.com/order/catalog/product/65-0865-14
BV480 Rat Anti-Mouse CD45: BD Biosciences, 752417, https://www.bdbiosciences.com/en-br/products/reagents/flow-cytometry-reagents/research-reagents/single-color-antibodies-ruo/BV480-Rat-Anti-Mouse-CD45.752417
FITC Rat Anti-CD11b(M1/70): BD Biosciences, 557396, https://www.bdbiosciences.com/en-au/products/reagents/flow-cytometry-reagents/research-reagents/single-color-antibodies-ruo/fitc-rat-anti-cd11b.557396
Ms Ly-6G PE-Cy7 1A8: BD Biosciences,560601, https://www.univ-bio.com/ly-6g/560601.html
CD206 (MMR) Monoclonal Antibody (MR6F3), PE: eBioscience, 12-2061-82, https://www.thermofisher.com/antibody/product/CD206-MMR-Antibody-clone-MR6F3-Monoclonal/12-2061-82
APC Rat Anti-Mouse CD4(RM4-5): BD Biosciences, 553051, https://www.bdbiosciences.com/en-nl/products/reagents/flow-cytometry-reagents/research-reagents/single-color-antibodies-ruo/apc-rat-anti-mouse-cd4.553051
Ms CD8a PE 53-6.7 100ug: BD Biosciences, 553032, https://www.bdbiosciences.com/en-pt/products/reagents/flow-cytometry-reagents/research-reagents/single-color-antibodies-ruo/pe-rat-anti-mouse-cd8a.553032
Ms CD44 BV421 IM7 50ug: BD Biosciences, 563970, https://www.bdbiosciences.com/en-nl/products/reagents/flow-cytometry-reagents/research-reagents/single-color-antibodies-ruo/bv421-rat-anti-mouse-cd44.563970
PE-Cy7 Rat Anti-Mouse CD62L(MEL-14): BD Biosciences, 560516, https://www.bdbiosciences.com/en-gb/products/reagents/flow-cytometry-reagents/research-reagents/single-color-antibodies-ruo/pe-cy-7-rat-anti-mouse-cd62l.560516
Fc Receptor Blocking Solution, Mouse: absin, abs9477-200T, https://www.absin.net/article-1445.html
APC anti-mouse CD11c: BioLegend, 117310, https://www.biolegend.com/en-gb/products/apc-anti-mouse-cd11c-antibody-1813?GroupID=BLG11937

FITC anti-mouse CD80: BioLegend, 104705, https://www.biolegend.com/en-gb/products/fitc-anti-mouse-cd80-antibody-41?GroupID=BLG274
Pacific Blue™ anti-mouse CD86: BioLegend, 105022, https://www.biolegend.com/en-gb/products/pacific-blue-anti-mouse-cd86-antibody-3122
Pacific Blue™ anti-mouse CD80 Antibody: BioLegend, 104723, https://www.biolegend.com/fr-fr/products/pacific-blue-anti-mouse-cd80-antibody-6267
PE anti-mouse CD86 Antibody: BioLegend, 159203, https://www.biolegend.com/nl-be/products/pe-anti-mouse-cd86-antibody-18945
FITC anti-mouse CD3: BioLegend, 100204, https://www.biolegend.com/nl-be/products/fitc-anti-mouse-cd3-antibody-45
anti-mouse CD69-FITC: BioLegend, 104505, https://www.biolegend.com/fr-fr/products/fitc-anti-mouse-cd69-antibody-264
PE anti-mouse CD4: BioLegend, 100512, https://www.biolegend.com/en-gb/products/pe-anti-mouse-cd4-antibody-482?GroupID=BLG4211
Pacific Blue™ anti-mouse CD8a: BioLegend, 100725, https://www.biolegend.com/de-at/products/pacific-blue-anti-mouse-cd8a-antibody-2856?GroupID=BLG279
APC anti-mouse IFN-γ: BioLegend, 505810, https://www.biolegend.com/fr-fr/products/apc-anti-mouse-ifn-gamma-antibody-993
FITC anti-mouse FOXP3: Invitrogen, #11-5773-82, https://www.thermofisher.com/antibody/product/FOXP3-Antibody-clone-FJK-16s-Monoclonal/11-5773-82

Eukaryotic cell lines

Policy information about cell lines and Sex and Gender in Research

Cell line source(s)	The B16-F10 cell line was purchased from IMMOCELL (Guangzhou, China). The CT26 (ATCC CRL-2638) and RAW264.7 cell lines were purchased from the American Type Culture Collection (ATCC).
Authentication	The cell line was authenticated
Mycoplasma contamination	Cell line were not contaminated by Mycoplasma.
Commonly misidentified lines (See ICLAC register)	No commonly misidentified cell line was used.

Palaeontology and Archaeology

Specimen provenance	*Provide provenance information for specimens and describe permits that were obtained for the work (including the name of the issuing authority, the date of issue, and any identifying information). Permits should encompass collection and, where applicable, export.*
Specimen deposition	*Indicate where the specimens have been deposited to permit free access by other researchers.*
Dating methods	*If new dates are provided, describe how they were obtained (e.g. collection, storage, sample pretreatment and measurement), where they were obtained (i.e. lab name), the calibration program and the protocol for quality assurance OR state that no new dates are provided.*

☐ Tick this box to confirm that the raw and calibrated dates are available in the paper or in Supplementary Information.

Ethics oversight	*Identify the organization(s) that approved or provided guidance on the study protocol, OR state that no ethical approval or guidance was required and explain why not.*

Note that full information on the approval of the study protocol must also be provided in the manuscript.

Animals and other research organisms

Policy information about studies involving animals; ARRIVE guidelines recommended for reporting animal research, and Sex and Gender in Research

Laboratory animals	Female BALB/c and C57BL/6 mice (6-8 weeks old) were purchased from Hangzhou Ziyuan Laboratory Animal Technology Co., Ltd. and housed in a specific pathogen-freefacility, All mice were maintained under controled conditions with a 12-hour light/dark cycle (lights on from 8:00 a.m. to 8:00 p.m.)constant temperature (18-22°C), and humidity (50-60%), with unrestricted access to standard chow and water.
Wild animals	The study did not involve wild animals.
Reporting on sex	Female mice were selected in this study. n ≥3 biologically independent mice per group.
Field-collected samples	The study did not involve samples collected from the field.
Ethics oversight	All animal experiments were performed in accordance with the National Institutes of Health animal care guidelines. Animal protocols were approved by the Institutional Animal Care and Use Committees on Animal Care (Nanjing University, IACUC-D2202146).

Note that full information on the approval of the study protocol must also be provided in the manuscript.

Clinical data

Policy information about clinical studies

All manuscripts should comply with the ICMJE guidelines for publication of clinical research and a completed CONSORT checklist must be included with all submissions.

Clinical trial registration	*Provide the trial registration number from ClinicalTrials.gov or an equivalent agency.*
Study protocol	*Note where the full trial protocol can be accessed OR if not available, explain why.*
Data collection	*Describe the settings and locales of data collection, noting the time periods of recruitment and data collection.*
Outcomes	*Describe how you pre-defined primary and secondary outcome measures and how you assessed these measures.*

Dual use research of concern

Policy information about dual use research of concern

Hazards

Could the accidental, deliberate or reckless misuse of agents or technologies generated in the work, or the application of information presented in the manuscript, pose a threat to:

No	Yes	
☐	☐	Public health
☐	☐	National security
☐	☐	Crops and/or livestock
☐	☐	Ecosystems
☐	☐	Any other significant area

Experiments of concern

Does the work involve any of these experiments of concern:

No	Yes	
☐	☐	Demonstrate how to render a vaccine ineffective
☐	☐	Confer resistance to therapeutically useful antibiotics or antiviral agents
☐	☐	Enhance the virulence of a pathogen or render a nonpathogen virulent
☐	☐	Increase transmissibility of a pathogen
☐	☐	Alter the host range of a pathogen
☐	☐	Enable evasion of diagnostic/detection modalities
☐	☐	Enable the weaponization of a biological agent or toxin
☐	☐	Any other potentially harmful combination of experiments and agents

Plants

Seed stocks	Not applicable
Novel plant genotypes	Not applicable
Authentication	Not applicable

ChIP-seq

Data deposition

☐ Confirm that both raw and final processed data have been deposited in a public database such as GEO.

☐ Confirm that you have deposited or provided access to graph files (e.g. BED files) for the called peaks.

Data access links<br>*May remain private before publication.*	*For "Initial submission" or "Revised version" documents, provide reviewer access links. For your "Final submission" document, provide a link to the deposited data.*
Files in database submission	*Provide a list of all files available in the database submission.*
Genome browser session<br>(e.g. UCSC)	*Provide a link to an anonymized genome browser session for "Initial submission" and "Revised version" documents only, to enable peer review. Write "no longer applicable" for "Final submission" documents.*

Methodology

Replicates	*Describe the experimental replicates, specifying number, type and replicate agreement.*
Sequencing depth	*Describe the sequencing depth for each experiment, providing the total number of reads, uniquely mapped reads, length of reads and whether they were paired- or single-end.*
Antibodies	*Describe the antibodies used for the ChIP-seq experiments; as applicable, provide supplier name, catalog number, clone name, and lot number.*
Peak calling parameters	*Specify the command line program and parameters used for read mapping and peak calling, including the ChIP, control and index files used.*
Data quality	*Describe the methods used to ensure data quality in full detail, including how many peaks are at FDR 5% and above 5-fold enrichment.*
Software	*Describe the software used to collect and analyze the ChIP-seq data. For custom code that has been deposited into a community repository, provide accession details.*

Flow Cytometry

Plots

Confirm that:

☒ The axis labels state the marker and fluorochrome used (e.g. CD4-FITC).

☒ The axis scales are clearly visible. Include numbers along axes only for bottom left plot of group (a 'group' is an analysis of identical markers).

☒ All plots are contour plots with outliers or pseudocolor plots.

☒ A numerical value for number of cells or percentage (with statistics) is provided.

Methodology

Sample preparation

1. Spleem Aseptically excise the spleen and immediately place it in cold complete RPMI-1640 medium (supplemented with 2% FBS). Maintain the spleen on ice or at 4°C throughout the procedure. Place the spleen in a 70 µm cell strainer positioned over a 50 mL Falcon tube. Add 2-5 mL of cold complete RPMI-1640 to the strainer and gently disrupt the spleen tissue by pressing it against the mesh using the plunger of a sterile 5 mL syringe (or a rubber pestle) in circular motions. Wash the cell strainer with an additional 2-3 mL of cold PBS or RPMI to maximize cell recovery. Collect the filtered cell suspension and centrifuge at 300-400 x g for 5 minutes at 4°C.
2. Bone Marrow: Aseptically excise the femurs and tibias. Remove excess tissue. Using a syringe with a 25G needle, flush the bone marrow from the bones with cold complete RPMI-1640 medium (supplemented with 2% FBS). Gently pipette the cell suspension to break up clumps. Place the cell suspension in a 70 µm cell strainer positioned over a 50 mL Falcon tube. Wash the cell strainer with an additional 2-3 mL of cold PBS to maximize cell recovery. Collect the filtered cell suspension and centrifuge at 300-400 x g for 5 minutes at 4°C.
3.Tumor-Draining Lymph Node (TDLN): Aseptically excise the TDLNs and immediately place them in cold complete RPMI-1640 medium (supplemented with 2% FBS). Maintain the lymph nodes on ice or at 4°C throughout the procedure. Place the lymph nodes in a 70 µm cell strainer positioned over a 50 mL Falcon tube. Add 2-5 mL of cold complete RPMI-1640 to the strainer and gently disrupt the lymph node tissue by pressing it against the mesh using the plunger of a sterile 5 mL syringe (or a rubber pestle) in circular motions. Wash the cell strainer with an additional 2-3 mL of cold PBS to maximize cell recovery. Collect the filtered cell suspension and centrifuge at 300-400 x g for 5 minutes at 4°C.
4. Peripheral Blood:
Collect peripheral blood samples into tubes containing an anticoagulant (e.g., EDTA or heparin) and immediately place them on ice or at 4°C. Dilute the whole blood with an equal volume of cold PBS or complete RPMI-1640 medium (supplemented with 2% FBS). Centrifuge the suspension at 300-400 x g for 5 minutes at 4°C to pellet the cells. Carefully discard the supernatant. Resuspend the cell pellet in Red Blood Cell (RBC) lysis buffer (according to the manufacturer's instructions) to remove erythrocytes, followed by quenching with cold complete RPMI-1640 or PBS. Centrifuge again at 300-400 x g for 5 minutes at 4°C to obtain the final leukocyte pellet.

5. Tumor Tissues:
Aseptically excise the tumor masses and remove any necrotic areas or connective tissue. Mince the tumor tissue into small pieces (approximately 1-2 mm^3) using sterile scissors and immediately place them in a digestion buffer (e.g., RPMI-1640 containing Collagenase IV and DNase I). Incubate the tissue at 37°C for 30–60 minutes with gentle agitation. After digestion, stop the reaction by adding cold complete RPMI-1640 medium (supplemented with 2% FBS) and place the samples on ice. Pass the digested tissue suspension through a 70 µm cell strainer positioned over a 50 mL Falcon tube. Gently aid dispersion by pressing remaining tissue fragments against the mesh using the plunger of a sterile 5 mL syringe. Wash the cell strainer with an additional 2-3 mL of cold PBS to maximize cell recovery. Collect the filtered cell suspension and centrifuge at 300-400 x g for 5 minutes at 4°C.

Instrument

Agilent NovoCyte

Software

Flow-cytometry analysis was performed in FlowJo v10.8.1 or NovoExpress v1.6.2

Cell population abundance

At least 10,000 relevant events were acquired for in vivo flow cytometry analysis.

Gating strategy

Representative flow cytometry gating strategy for immune cell phenotyping. General gating workflow: Cells were initially gated on lymphocytes based on forward scatter (FSC) and side scatter (SSC) characteristics, followed by doublet exclusion using FSC-H/FSC-A and SSC-H/SSC-A plots. Red arrows indicate the sequential gating path. Sample sources and specific staining details are described in the Methods section.
(a) Splenocyte-derived T cell subsets: Live CD45+ cells were gated on CD3+ T cells, then subdivided into CD4+ and CD8+ populations. Memory phenotypes were defined as central memory (TCM:CD44+CD62L+) and effector memory (TEM: CD44+ CD62L-).
(b) Lymph node-derived dendritic cell (DC) subsets: CD11c+ cells were gated on single cells, with activation status evaluated by CD80 and CD86 co-expression.
(c) Bone marrow-derived macrophage subsets: Live CD45+ cells were gated on CD11b+ F4/80+ macrophages, with polarization assessed by CD86 (M1-like) and CD206 (M2-like) expression.
(d) Splenocyte-derived Treg cells: Live $CD45^+$ cells were first gated on singlets, then on $CD4^+$ T cells. Regulatory T cells (Treg) were defined as $CD25^+FoxP3^+$ cells within the $CD4^+$ population.
(e) Lymph node-derived total T cell subsets: CD3+ cells were gated on single cells, then separated into CD4+ and CD8+ populations.
(f) Lymph node-derived early-activated T cell subsets: Early activation was assessed by directly gating CD69+CD4+ and CD69 +CD8+ T cells using CD69 versus CD4 or CD69 versus CD8 dual-parameter plots.
(g) Splenocyte-derived cytotoxic T cell function: After gating on singlets and CD3+ T cells, CD8+ T cells were identified and further analyzed for intracellular IFN-γ expression following stimulation.
(h) Gating strategy for Neutrophils: After excluding debris and doublets, live CD45+ leukocytes were identified. Neutrophils were then defined and gated as CD11b+Ly6G+ cells within the leukocyte population.

☒ Tick this box to confirm that a figure exemplifying the gating strategy is provided in the Supplementary Information.

Magnetic resonance imaging

Experimental design

Design type

Indicate task or resting state; event-related or block design.

Design specifications

Specify the number of blocks, trials or experimental units per session and/or subject, and specify the length of each trial or block (if trials are blocked) and interval between trials.

Behavioral performance measures

State number and/or type of variables recorded (e.g. correct button press, response time) and what statistics were used to establish that the subjects were performing the task as expected (e.g. mean, range, and/or standard deviation across subjects).

Acquisition

Imaging type(s)

Specify: functional, structural, diffusion, perfusion.

Field strength

Specify in Tesla

Sequence & imaging parameters

Specify the pulse sequence type (gradient echo, spin echo, etc.), imaging type (EPI, spiral, etc.), field of view, matrix size, slice thickness, orientation and TE/TR/flip angle.

Area of acquisition

State whether a whole brain scan was used OR define the area of acquisition, describing how the region was determined.

Diffusion MRI ☐ Used ☐ Not used

Preprocessing

Preprocessing software

Provide detail on software version and revision number and on specific parameters (model/functions, brain extraction, segmentation, smoothing kernel size, etc.).

Normalization

If data were normalized/standardized, describe the approach(es): specify linear or non-linear and define image types used for

transformation OR indicate that data were not normalized and explain rationale for lack of normalization.

Normalization template: Describe the template used for normalization/transformation, specifying subject space or group standardized space (e.g. original Talairach, MNI305, ICBM152) OR indicate that the data were not normalized.

Noise and artifact removal: Describe your procedure(s) for artifact and structured noise removal, specifying motion parameters, tissue signals and physiological signals (heart rate, respiration).

Volume censoring: Define your software and/or method and criteria for volume censoring, and state the extent of such censoring.

Statistical modeling & inference

Model type and settings: Specify type (mass univariate, multivariate, RSA, predictive, etc.) and describe essential details of the model at the first and second levels (e.g. fixed, random or mixed effects; drift or auto-correlation).

Effect(s) tested: Define precise effect in terms of the task or stimulus conditions instead of psychological concepts and indicate whether ANOVA or factorial designs were used.

Specify type of analysis: ☐ Whole brain ☐ ROI-based ☐ Both

Statistic type for inference (See Eklund et al. 2016): Specify voxel-wise or cluster-wise and report all relevant parameters for cluster-wise methods.

Correction: Describe the type of correction and how it is obtained for multiple comparisons (e.g. FWE, FDR, permutation or Monte Carlo).

Models & analysis

n/a	Involved in the study
☐	☐ Functional and/or effective connectivity
☐	☐ Graph analysis
☐	☐ Multivariate modeling or predictive analysis

Functional and/or effective connectivity: Report the measures of dependence used and the model details (e.g. Pearson correlation, partial correlation, mutual information).

Graph analysis: Report the dependent variable and connectivity measure, specifying weighted graph or binarized graph, subject- or group-level, and the global and/or node summaries used (e.g. clustering coefficient, efficiency, etc.).

Multivariate modeling and predictive analysis: Specify independent variables, features extraction and dimension reduction, model, training and evaluation metrics.
